# Septal and Hippocampal Neurons Contribute to Auditory Relay and Fear Conditioning

**DOI:** 10.3389/fncel.2018.00102

**Published:** 2018-04-16

**Authors:** Cuiyu Xiao, Yun Liu, Jian Xu, Xiong Gan, Zhongju Xiao

**Affiliations:** Key Laboratory of Psychiatric Disorders of Guangdong Province, Department of Physiology, School of Basic Medical Sciences, Southern Medical University, Guangzhou, China

**Keywords:** auditory cortex, entorhinal cortex, hippocampus, lemniscal pathway, non-lemniscal pathway

## Abstract

The hippocampus has been thought to process auditory information. However, the properties, pathway, and role of hippocampal auditory responses are unclear. With loose-patch recordings, we found that hippocampal neurons are mainly responsive to noise and are not tonotopically organized. Their latencies are shorter than those of primary auditory cortical (A1) neurons but longer than those of medial septal (MS) neurons, suggesting that hippocampal auditory information comes from MS neurons rather than from A1 neurons. Silencing the MS blocks both hippocampal auditory responses and memory of auditory fear conditioning trained with noise and tone. Auditory fear conditioning was associated with some cues but not with a specific frequency of sound, as demonstrated by animals trained with noise, 2.5-, 5-, 10-, 15-, or 30-kHz tones, and tested with these sounds. Therefore, the noise responses of hippocampal neurons have identified a population of neurons that can be associated with auditory fear conditioning.

## Introduction

The hippocampus plays a critical role in learning and memory. Many behavioral experiments have been designed using sound as a cue to explore the functions of the hippocampus, such as sound discrimination (Vinogradova, 1975; O’Keefe and Nadel, 1978; Sakurai, 2002; Itskov et al., 2012; Vinnik et al., 2012), auditory recognition (Vinogradova, 1975; O’Keefe and Nadel, 1978; Sakurai, 1990) and auditory fear conditioning (O’Keefe and Nadel, 1978; LeDoux, 2000; Moita et al., 2003; Buccafusco, 2009). In auditory fear conditioning, the hippocampus has been thought to process auditory information carried by conditioned stimuli (CS, tone) and to associate it with an aversive unconditioned stimulus (US, electric foot shock), resulting in a behavioral conditioned fear response (freezing) to the CS (Bouton and Bolles, 1980; Fanselow, 1980). In the studies of the hippocampus, various sounds, such as tone bursts (Sacchetti et al., 1999; Maren and Holt, 2004; Czerniawski et al., 2012; Robinson and Bucci, 2012; Donzis et al., 2013; Kaifosh et al., 2013), clicks (O’Keefe and Nadel, 1978; McHugh et al., 2013) and noise bursts (Moita et al., 2003, 2004; Kaifosh et al., 2013) have been used as CS for auditory fear conditioning. However, it is unclear whether hippocampal neurons respond to the sounds used as CS, where the auditory information processed by these neurons comes from, and how their sound responses are related to behavioral responses, such as freezing.

What are the properties of hippocampal neurons responses to sound? The hippocampus is a well-known structure of the limbic system. The neurons in the limbic system, such as in the amygdala, striatum, cingulate cortex (Vinogradova, 1975; Chen et al., 2014) and septum (Vinogradova, 1975; Mercer and Remley, 1979; Zhang et al., 2011), briefly respond to noise bursts. Neurons in the amygdala and striatum that respond to tone bursts are tonotopically organized (Bordi and LeDoux, 1992; Chen et al., 2012). Hippocampal neurons also respond to noise bursts (Vinogradova, 1975; O’Keefe and Nadel, 1978; Chen et al., 2014; Wang et al., 2017), clicks (Vinogradova, 1975; Brankack and Buzsaki, 1986; Moxon et al., 1999) and tone bursts (Vinogradova, 1975; O’Keefe and Nadel, 1978; Miller and Freedman, 1995; Krause et al., 2003; Dissanayake et al., 2008). These studies were performed by recording auditory evoked potentials from anesthetized (Miller and Freedman, 1995; Krause et al., 2003; Dissanayake et al., 2008) or awake (Brankack and Buzsaki, 1986; Ruusuvirta et al., 1995; Moxon et al., 1999) animals and by extracellularly recording single unit responses in both anesthetized (Krause et al., 2003) and awake (Moita et al., 2003) rats. A recent study using an *in vivo* whole-cell recording technique showed that the auditory responses of most hippocampal CA1 neurons involve hyperpolarization following the onset of a tonal stimulus, but only in awake animals (Abe et al., 2014). Hyperpolarization itself does not evoke action potentials, but its rebound depolarization does. Thus, the data obtained by whole-cell recordings appear different from those that are extracellularly recorded. Therefore, the response properties of hippocampal neurons to sound remain to be further studied in anesthetized and awake animals.

Where is the hippocampal auditory information from? The transmission of auditory information to the hippocampus has been thought to come through two major pathways. One is the lemniscal pathway, which is the ascending auditory pathway from the cochlear nucleus to the auditory cortex (AC) via the lateral lemniscus (Syka and Masterton, 1988; Webster et al., 1992; Winer and Schreiner, 2011); beyond the AC, auditory signals go to the association cortex, entorhinal cortex (EC) and hippocampus (Vinogradova, 1975; Steward, 1976; Germroth et al., 1989). The other is the non-lemniscal pathway, which consists of the brainstem auditory nuclei, brainstem reticular nucleus, medial septum (MS) and hippocampus (Vinogradova, 1975; Baisden et al., 1984; Moxon et al., 1999). However, these pathways have not yet been functionally confirmed. Lemniscal neurons are tonotopically organized (Aitkin and Webster, 1972; Webster et al., 1992; Winer and Schreiner, 2011), narrowly tuned to a specific single frequency, and respond to repetitive tonal stimuli with fidelity. However, non-lemniscal neurons are not tonotopically organized, but are broadly frequency tuned, are able to rapidly adapt to repetitive identical stimuli (Aitkin and Webster, 1972; Phillips and Irvine, 1979), and are involved in multisensory integration, temporal pattern recognition, and certain forms of learning (Webster et al., 1992). Therefore, detailed studies of the response properties of hippocampal neurons can tell us which pathway is predominantly operating. Auditory responses of some hippocampal neurons to a conditioned sound are enhanced by auditory fear conditioning training (O’Keefe and Nadel, 1978; Moita et al., 2003). However, this finding does not mean that hippocampal neurons respond to a conditioned sound. In other words, if some hippocampal neurons respond to some characterized sound, whether this sound is a more efficient conditioned sound in auditory fear conditioning in evoking behavioral responses, such as freezing, remains to be determined. Thus, what is the role of the responses of hippocampal neurons?

In the current study, we mainly investigated the properties, pathway, and role of hippocampal auditory responses, respectively. The goal of this study was to obtain the answers to the above three issues using loose-patch recordings, combined with reversible inactivation of the lemniscal or non-lemniscal pathway and auditory fear conditioning. We found that hippocampal neurons activated by the MS respond only to noise stimuli in awake subjects and the noise responses of hippocampal neurons have identified a population of neurons that can be associated with behavior, which is independent of the frequency of conditioned sound. These findings provided new insights for further studies of fear memory in the limbic system.

## Materials and Methods

### Animal Preparation

Our experimental protocols were approved by the Animal Care and Use Committee of Southern Medical University, Guangzhou, China. For the experiments, we used 180 C57BL/6 mice (females, 4–6 weeks, 14–20 g, housed under a 12-h light/dark cycle) with normal hearing in total. They were bred in the Experimental Animal Center of Southern Medical University.

As in our previous experiments (Xiong et al., 2013; Huang et al., 2015; Wang et al., 2017), sodium pentobarbital (60–70 mg/kg, i.p., Sigma, United States) and atropine sulfate (0.25 mg/kg, s.c., Nandao, Hainan, China) were injected into each mouse for anesthesia and inhibition of respiratory secretions, respectively. The effects of anesthesia on each mouse were examined using the pedal withdrawal reflex and were maintained by supplemental doses (13 mg/kg) of pentobarbital during the surgery. Each mouse’s skull was exposed under a stereotaxic apparatus. A 1.5-cm-long nail was mounted on the top of the skull for the electrophysiological experiments, and for pharmacological manipulation, one (occasionally two) 0.5-cm-long guide cannula (O.D. 0.41 × I.D. 0.25 mm) was embedded in the skull over the A1 (3.0 mm posterior to bregma, 4.2 mm lateral to midline) or the MS: (0.8 mm anterior to bregma, 1.0 mm lateral to midline, at an angle of 13° in the lateral direction) with dental cement. A stainless steel obturator (O.D = 0.2 mm) was inserted into the guide cannulas to prevent jamming. After the surgery, local anesthetic (lidocaine hydrochloride) and antibiotic ointment (furacin) were applied to the surgical wound, and each mouse was kept alone for recovery at least 7 days.

Each mouse with the head-fixed metal post for electrophysiological recordings was trained on a rotatable plate to adapt for the electrophysiological recording that was started after the 7-day recovery period. To fix the mouse’s head, the nail mounted on the skull was attached to a small metal rod on an anti-vibration table (TMC, Peabody, MA, United States) with setscrews. Training was repeated approximately 1–2 h/day (consecutively on days 5–8) until the mouse learned to stay quiet and run freely in the center of the plate (Abe et al., 2014). One day before the electrophysiological recordings, the mouse was anesthetized with sodium pentobarbital (60–70 mg/kg, i.p.), returned to the plate and fixed on the table. A small window (1 mm × 1 mm) was made in the skull over an intended recording site without removing the dura. The exposed brain was covered with Vaseline to prevent desiccation. An Ag/AgCl reference electrode was placed under the prefrontal bone.

### Loose-Patch Recordings in Awake Mice

All electrophysiological recordings were made in a soundproof room maintained at 24–26°C. The awake animal’s head was immobilized by fixing the metal post mounted on the skull to a small metal rod on the anti-vibration table, while its body was able to run freely on the rotatable plate. The Vaseline and dura of the window were removed and impaled, respectively. Loose-patch recordings were performed with a MultiClamp 700B amplifier (Axon Instruments/Molecular Devices, Sunnyvale, CA, United States) under voltage-clamp mode without applying a holding voltage as previously reported (Turner et al., 2005; Wu et al., 2008; Xiong et al., 2013). A glass pipette (tip ∼1 μm in diameter, 5–7 MΩ impedance) filled with artificial cerebrospinal fluid (ACSF; in mM: 124 NaCl, 2.5 KCl, 1.2 NaH_2_PO_4_, 25 NaHCO_3_, 1 MgCl_2_, 2 CaCl_2_, 20 glucose, and 0.5% biocytin, pH 7.2) was vertically inserted into the recording sites with a micromanipulator (Siskiyou Inc., Grants Pass, OR, United States). The patch pipette with a positive pressure (0.5–1 Psi) was advanced in 1-μm steps until the resistance changed (10–20 MΩ). Subsequently, the positive pressure was withdrawn, and a negative pressure (∼0.3 Psi) was applied. When the system resistance reached 0.2–1 GΩ, loose-patch recording of the neuron was performed. Neuronal signals (spikes) were amplified and filtered with a 300–3,000 Hz band-pass filter. Current spikes were sorted using the BrainWare software (Version 9.21, Tucker-Davis Technologies, Alachua, FL, United States). Data were recorded and stored in DAM and SRC files for off-line analysis. Each electrophysiological recording session lasted approximately 10–30 min. Between the recording sessions, 5% sucrose drops were given to the mouse through a pipe.

The recording sites of EC, CA3, CA1, MS, and A1 were determined based on the coordinates (posterior to bregma, lateral to midline, and below the brain surface in mm) of the Allen Mouse Brain Atlas (Lein et al., 2007). EC: 4.0–5.0, 3.0–3.5, 1.5–3.5; CA3: 2.5–3.5, 3.0–3.5, 1.5–3.5; CA1: 2.5–3.0, 2.0–2.8, 1.2–1.5; MS: -0.6–-1.1, 1.0, 3.5–5.0; and A1: 2.5–3.5, 4.0–4.5, 0.2–0.8. Each animal’s head was fixed as in the atlas for the mouse brain, except during the EC and MS recording sessions, in which the animal’s head was laterally rotated 9 and 13°, respectively. Histological examinations verified that all electrode tips were located within the target area.

### Juxtacellular Labeling and Biocytin Staining

To reveal the somatic and dendritic morphologies and the locations of the recorded neurons, juxtacellular labelings were performed. Once a loose-patch configuration was completed, we applied current pulses of 3–10 nA for 300 ms ON and 300 ms OFF for up to 20 min, as in previous papers (Pinault, 1996; Turner et al., 2005; Wu et al., 2008). We monitored the noise-evoked spikes to ensure that there was neither damage to the cell nor drifting of the recording pipette. Then, the mouse received an overdose of pentobarbital sodium (100 mg/kg, i.p.) and was perfused transcardially with physiological saline (0.9%) and fixative [4% paraformaldehyde (PFA) in 0.01 M phosphate-buffered saline (PBS), pH 7.4] immediately. The brain was removed and post-fixed with 4% PFA for 24 h at 4°C. It was coronally sectioned into 50-μm-thick sections with a freezing microtome (Leica CM 1950, Nussloch, Germany) after 24 h of immersion in 20 and 30% sucrose for cryoprotection. The free-floating sections were washed three times with PBS for 10 min each time. To increase the permeability of the antibody in the cell membrane, the sections were incubated with 0.3% Triton X-100 for 1 h. After rinsing three times for 10 min each with PBS, the sections were incubated with Streptavidin-Cy3 (1:200, molecular probes, catalog no.43–4315, Eugene, OR, United States) and bovine serum albumin (5%, Boster, AR0004, Wuhan, China) at room temperature for 4 h. Aluminum foil was used to shield the sections from light. The sections were then washed with distilled water and transferred to subbed slides. After drying, the sections were stained with 0.25 μg/mL 4′, 6-diamidino-2-phenylindole (DAPI) to visualize the neuronal cell nuclei. The slides were examined using a confocal microscope (Nikon, A1R, Japan).

### Sound Stimulation

As in our previous experiments (Xiong et al., 2013; Huang et al., 2015; Wang et al., 2017), acoustic stimuli (1–50 kHz noise and 2–64 kHz tone bursts, 50 ms in duration and 5 ms rise/fall time) were generated using a Tucker-Davis Technologies System 3 (TDT 3, Tucker-Davis Technologies, Alachua, FL, United States). The intensities were controlled by a programmable attenuator (PA5). The synthesized signals from RX6 were amplified using an electrostatic speaker driver (ED1) and delivered through a loudspeaker (ES1, frequency range 2–110 kHz) placed 15 cm in front of the mouse. The loudspeaker was calibrated with 1/8- and 1/4-inch microphones and an amplifier (Brüel and Kjaer 4138, 4135 and 2610, Naerum, Denmark) at the beginning of the experiment. The amplitudes of the tones and noise bursts were expressed as the sound pressure level (SPL, 0 dB re 20 μPa). The tone bursts varied in frequency (2–64 kHz, at 0.1 octave steps) and amplitude (0–90 dB SPL, 10 dB steps) for the frequency-amplitude (F-A) scans. Noise bursts varied in amplitude (0–90 dB SPL, at 10 dB steps) during the amplitude scan (A-scan). The sound parameters of the F-A and A-scans were controlled by the Brain Ware software through a computer and were presented pseudo-randomly at a rate of 1/s. The F-A scans were repeated 3–5 times to map a complete receptive field. The A-scan was repeated 20–30 times to obtain an array of peri-stimulus time histograms (PSTH).

### Inactivation of the A1 and MS

Lidocaine hydrochloride [20 mg/mL, in physiological saline containing 5% biotinylated dextran amines (BDA, Invitrogen, D1828, United States)] (Martin and Ghez, 1999) and muscimol (1 mg/mL in physiological saline, Tocris Bioscience, catalog no. 0289, United Kingdom) (Martin and Ghez, 1999) were used in the electrophysiological and behavioral experiments, respectively. To inactivate the A1 and MS, the obturators in the guide cannula mounted on the mouse’s skull was screwed off and substituted with an infusion cannula (O.D. 0.21 mm × I.D. 0.11 mm) connected to a silicone tube that was connected to a microsyringe (Hamilton 5 μL, Model 7105, Reno, NV, United States). The infusion cannula penetrated 0.4 or 4.0 mm from the brain surface through the dura mater into the A1 or MS. The drugs (100–150 nL in total, gauged with mineral oil) were injected at a rate of 0.2 μL/min using a Hamilton microsyringe that was controlled by a hydraulic pump (Tehovnik and Sommer, 1997). After the experiments, the diffusion ranges of the injected drugs were histologically examined.

### Histology

After the pharmacological experiments, the mice were euthanized with an overdose of sodium pentobarbital (100 mg/kg, i.p.) and perfused transcardially with physiological saline, followed by 4% PFA, as described above. The brains were post-fixed in 4% PFA overnight and cut into 50-μm-thick coronal sections with a freezing microtome. Then, the sections were mounted on the slides. The slides were examined using a confocal microscope to evaluate the cannula placements and drug infiltration.

### Behavioral Procedure

Auditory fear conditioning was performed for the sound characteristics, anesthesia and pharmacological manipulation experiments. Auditory fear conditioning includes trace fear conditioning and delay fear conditioning. Fear conditioning is an associative learning task in which subjects are presented with a neutral CS (such as a tone) paired with an aversive US (footshock). The subject quickly learns that the CS predicts the US. As the predictive relationship between the CS and US is learned, subsequent presentations of the CS alone elicit a conditional response, such as freezing. Trace fear conditioning has a temporal gap between a tone CS and an aversive electrical footshock US, whereas delay fear conditioning does not. The hippocampus is required for fear conditioning (Raybuck and Lattal, 2011). Therefore, here, we used a trace fear conditioning model to train the animals. In the pharmacological manipulation experiments, 48 mice were randomly and equally divided into eight subgroups (A1- and MS-injected saline or muscimol for noise or 2.5-kHz tone burst training, respectively). Each subgroup was trained with noise or a 2.5-kHz tone burst as the CS 10–15 min after drug injection. In the anesthesia experiments, two groups, awake and anesthetized, were trained and tested with noise burst. Regarding the sound characteristic experiments, a schematic diagram of the training protocol is shown (Supplementary Figure S4). Forty-two mice were equally divided into seven subgroups for training, and each mouse was conditioned to one kind of auditory stimulus (no-sound, noise, 2.5-, 5-, 10-, 15-, or 30-kHz tones) (**Figure 9**) but tested with multiple sounds (noise, 2.5-, 5-, 10-, 15-, and 30-kHz tones) 24 h after the training. All of the auditory fear conditioning experiments included habituation, training and testing.

On day 1 (habituation), each mouse was brought into the training room and placed in the conditioning chamber (Chamber A) (Habitest, Coulbourn Instruments, Holliston, MA, United States) for 20 min of habituation and was then returned to its home cage. Chamber A consisted of a grid floor that delivered a scrambled electric foot shock as the US. The walls of chamber A were made of opaque black plastic sheets. In the pharmacological intervention experiments, drug injectors were passed without infusion, and each mouse was acclimated to handling before habituation (Sacco and Sacchetti, 2010).

On day 2 (training), auditory trace fear conditioning took place in chamber A. Each mouse was trained in a single session that consisted of four trials. Each trial consisted of an auditory CS followed by a foot shock US. The loudspeaker in chamber A was replaced with another loudspeaker (ES1, 2–110 kHz connected to a TDT 3 with an RX6, a PA5 and an ED1), which was located 20 cm above the floor. After a 3-min habituation period in Chamber A, the mouse was delivered a 30-s CS (noise, 2.5-, 5-, 10-, 15-, or 30-kHz tone bursts, 80 dB SPL), followed by a 20-s trace interval and then by a 1-s presentation of the US (0.5 mA current) as a trial, as reported by Sacco and Sacchetti (2010) and Kaifosh et al. (2013). Four conditioned stimuli were delivered with 80-s intervals. After the last trial, each mouse was returned to the home cage. Between the experiments, the shock grids and floor trays were cleaned with 70% ethyl alcohol, the bedding was replaced with new material, and the chamber walls were cleaned with wet paper towels.

In the anesthetized training group, each mouse was anesthetized with sodium pentobarbital (60–70 mg/kg, i.p.). The overall training protocol was the same as described above in the awake mice. In the pharmacological intervention experiments, muscimol was injected into the MS or bilateral A1 of the mouse through the implanted cannula. The injections were performed 10 min before the auditory fear conditioning. After injection, the injector was kept for 3 min (Sacco and Sacchetti, 2010).

On day 3 (testing), retention of fear memory was tested in the testing chamber (Chamber B) by another experimenter. Chamber B was the same size as the chamber A, but its floor and walls were different to avoid conditioned fear behavior in response to contextual cues (Sacco and Sacchetti, 2010). The floor of Chamber B was covered with bedding materials, and its walls were made of brown planks. For each test, the mouse was put into the chamber B for 3 min to habituate and then given a CS (noise, 2.5-, 5-, 10-, 15-, or 30-kHz tone bursts) for 3 min without the US. For the sound characteristic experiments, each mouse underwent six different tests. The interval between each test was 1 h to reduce the cross-interactions between two tests and to allow each mouse to have a sufficient rest for the next test. To reduce the effect of test sound sequence, the sound tested randomly in a counterbalanced order. The conditioned response was freezing. The freezing response was defined as no somatic mobility, except for respiratory movements, and was scored automatically by using Graphic State 4 (Coulbourn Instruments, Holliston, MA, United States). The retention of the freezing response was tested on days 2, 4, 7, 14, and 28.

### Statistics and Data Analysis

The data analysis was performed with custom-developed software (MATLAB2012b, MathWorks). The analysis performers were partially blind to the conditions of the experiments, as the data from all the recorded neurons were first pooled together for randomized batch processing. Response latency was determined from PSTH as the time point when the activity exceeded the average baseline (measured in the time window of 900–1000 ms after stimulus onset) by three standard deviations of baseline fluctuations. Response threshold was the minimum stimulus intensity that could induce a sound-evoked spike in a neuron. The evoked spike rate was calculated from the spike counts within a 100-ms time window starting from the stimulus onset and was compared with the baseline firing rate during the 900–1000-ms sampling period. The bin size of the PSTHs is 1-ms. The percentages of freezing were expressed as the duration of the freezing response divided by that of the CS presentation.

Statistical analyses were performed in SPSS (Version 19, IBM). Datasets were first tested for normal distribution (Shapiro–Wilk test) and equal variances (Levene’s test). For two correlated group comparisons, a paired *t*-test was used. For continuous measurement data, repeated-measures analysis of variance (ANOVA) was applied to test the significance. For three or more group comparisons, a one-way ANOVA was used with the Scheffe *post hoc* test for multiple comparisons. For the frequency characteristics of the behavioral experiments, three-way analysis of covariance (ANCOVA) was applied with the Student–Newman–Keuls *post hoc* test for multiple comparisons. The experiments and data analysis were completed by different people (in section “Auditory Fear Conditioning Independent of the Frequency of Conditioned Sound”). For non-normally distributed data, a Mann–Whitney test was applied to assess significance. The confidence level was taken at 95% (*P* < 0.05). The summarized data are presented in the figures as the mean ± SEM, unless otherwise specified. The data plotting was carried out using the Origin software (version 8, OriginLab).

## Results

### Neurons in the Hippocampus (CA3, CA1, and EC) Responded to Noise Burst Stimuli Only in Awake Mice

**FIGURE 1:**
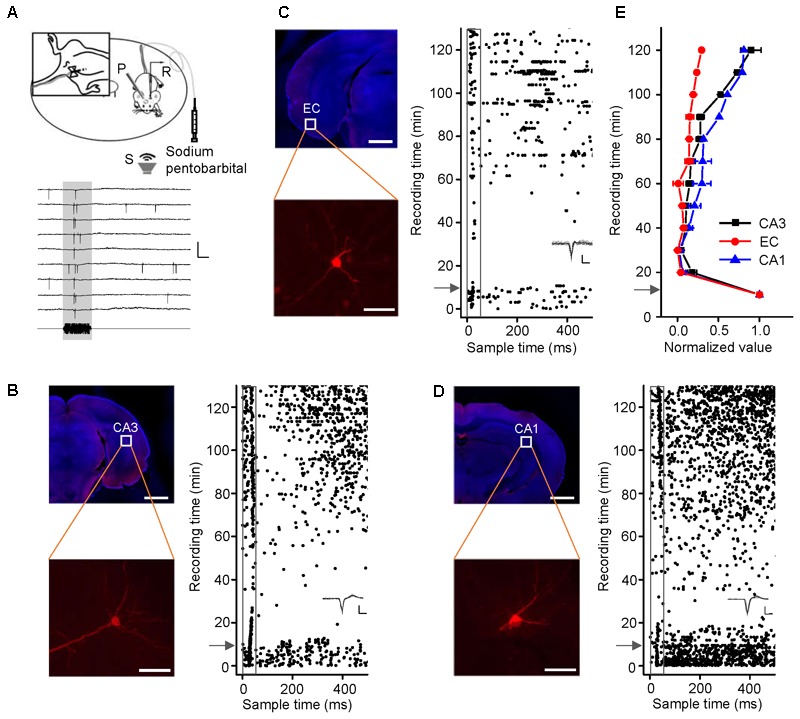
Noise-evoked responses of hippocampal neurons in awake and pentobarbital-anesthetized mice. **(A)** Top, diagram of loose-patch recordings in awake, head-fixed mice. The upper left panel shows an indwelling needle for intraperitoneal injection of sodium pentobarbital. R, recording electrode; P, metal post for head fixation; S, sound source. Bottom, 275-ms raw traces showing that the noise stimuli evoked spikes. Scale bars: *y* = 2 mV, *x* = 20 ms; the gray shade indicates the duration of the sound stimulation (50 ms). **(B–D)** Noise-evoked responses (right panels, raster plots) of neurons (left panels, Scale bar, 1000 μm; Enlarged figure, Scale bar, 50 μm) in the CA3 **(B)**, entorhinal cortex (EC) **(C)**, and CA1 **(D)** regions of mice before and after sodium pentobarbital injection (arrows). The gray boxes indicate 50-ms-long acoustic stimulation. Inset: 20 randomly selected superimposed spike waveforms. Scale: 40 pA, 0.5 ms. **(E)** Changes in the normalized responses of neurons in the CA3 (black, *n* = 4), EC (red, *n* = 3), and CA1 (blue, *n* = 3) regions after a pentobarbital injection. Each point represents a spike rate normalized by that measured before the injection.

CA3 is the intermediate link in the hippocampus. Loose-patch recordings of single-cell responses to acoustic stimuli were first performed in the CA3 regions of mice anesthetized with sodium pentobarbital (60–70 mg/kg, i.p.). However, we could detect neither evoked potentials nor spontaneous discharges. Thus, we performed the identical experiments in awake mice (**Figure 1A**, *the upper panel*). An example of a neuron spike raw trace was extracted (**Figure 1A**, *the lower panel*). A patched CA3 cell was juxtacellularly labeled (**Figure 1B**, *left panel*). Loose-patch recordings with loose seals (0.2–1 GΩ) allowed spikes only from the patched cell to be recorded. The cell discharged strongly in response to a noise burst stimulus with a bandwidth of 1–50 kHz at 90 dB SPL, decibels in sound pressure level (**Figure 1B**, *right panel, below the arrow*). To confirm the effect of anesthesia on CA3 neurons, we injected sodium pentobarbital (60–70 mg/kg, i.p.) with a needle indwelled in each animal’s peritoneal cavity (**Figure 1A**, *left inset*). The auditory response and spontaneous discharges both faded away within 1 min of the injection, and they gradually recovered over the 50 min thereafter (**Figure 1B**, *right panel, above the arrow*). To examine whether sodium pentobarbital also silenced other hippocampal neurons, auditory responses were recorded from neurons in the EC (**Figure 1C**) and CA1 (**Figure 1D**). The patched neurons in both areas (**Figures 1C,D**, *left panels*) exhibited changes in their responses and spontaneous discharges (**Figures 1C,D**, *right panels*) similar to those in the CA3 neurons (**Figure 1B**, *n = 4*). That is, the effects of anesthesia on the CA3 neurons [repeated-measures ANOVA, *F*_(2,4)_ = 2.307, *P* = 0.216] were similar to those in the EC (*n* = 3), and CA1 (*n* = 3) (**Figure 1E**).

**FIGURE 2:**
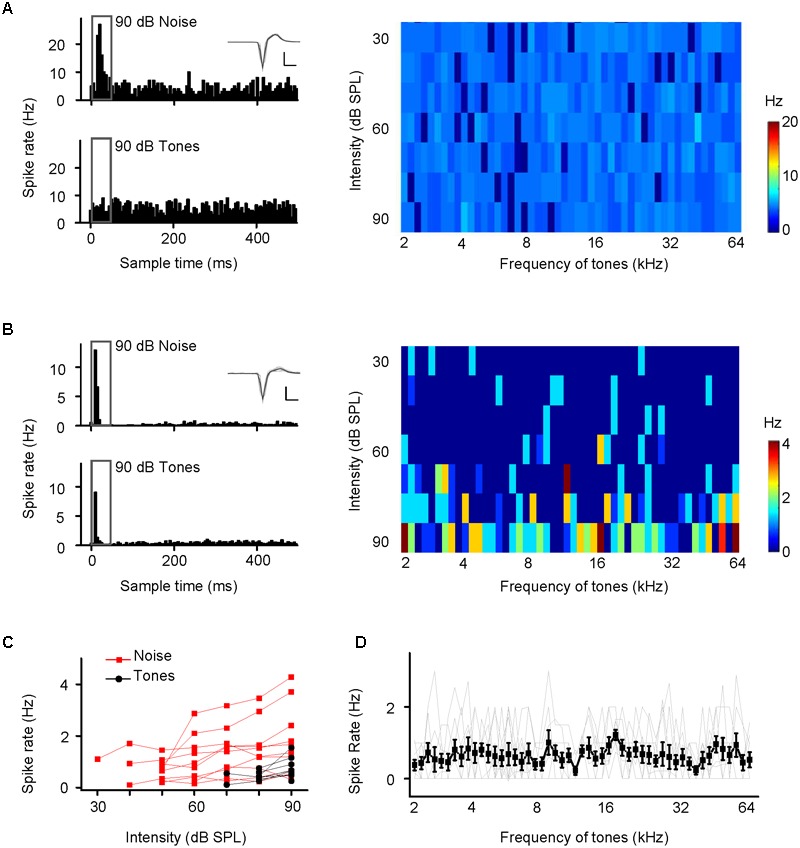
Two examples of hippocampal neuron responses to noise and tone stimuli. **(A,B)** Responses of CA3 neurons to noise bursts alone **(A)** and to noise and tone bursts **(B)**. Upper left panel, PSTH displaying the responses to noise at 90 dB SPL, extracted from the amplitude scans. Lower left panel, PSTH displaying the responses to all test tones at 90 dB SPL extracted from the responses to the frequency-amplitude scans displayed on the right color map, which depicts instantaneous discharge rates. Inset: 20 randomly selected superimposed spike waveforms. Scale: 60 pA, 0.5 ms. **(C)** A small number of neurons in the EC (*n* = 5), CA3 (*n* = 2), and CA1 (*n* = 3) that can respond to tone bursts. Spike rate-intensity functions of 10 neurons in response to both noise (red) and tone (black) stimulation. **(D)** Spike rate-frequency functions of the 10 neurons, individual (gray lines) and mean ± SEM (black line). The spike rate at each frequency was based on the responses at all intensities.

Noise-evoked discharges were recorded in approximately 15% of the neurons in the EC (65/382), CA3 (93/584), and CA1 (69/459). Most of them responded only to noise bursts and not to tone bursts as shown by an example CA3 neuron; the cell exhibited strong spike responses to noise bursts (**Figure 2A**, *upper left panel*, PSTH) but did not tune to any tone bursts, as shown by PSTH (**Figure 2A**, *lower left panel*) and a frequency amplitude (F-A) scan (**Figure 2A**, *right panel*). Similar responses were observed in the EC and CA1 regions, neurons in the EC and CA1 also only responded to noise bursts and almost not to tone bursts (Supplementary Figures S1, S2), so this kind of neuron could be in the majority population in the recording regions of EC (60/65), CA3 (91/93), and CA1 (66/69). However, there are a small number of neurons in the EC (5/65), CA3 (2/93), and CA1 (3/69) that can respond to tone bursts. As shown by an example CA3 neuron, this cell exhibited strong spike responses to both noise bursts (**Figure 2B**, *upper left panel*, PSTH) and tone bursts (**Figure 2B**, *lower left panel*, PSTH), but its tonal receptive fields (TRFs) were obscure, although it responded to certain frequencies at high stimulus levels (80–90 dB SPL) (**Figure 2B**, *right panel*). All of these tone-responsive neurons had low thresholds for noise bursts (30–60 dB SPL, **Figure 2C**, red lines), which were much lower than those for tone bursts 70–90 dB SPL (**Figure 2C**, black lines). The tone responses did not show any preference in terms of frequency [**Figure 2D**, one-way ANOVA, *F*(50,459) = 0.874, *P* = 0.715]. In the present study, both the lateral and the medial entorhinal cortices (ECl and ECm, respectively) were recorded (Supplementary Figure S3A). In regard to CA3 and CA1, the main recording sites were made in the pyramidal cell layer (Supplementary Figures S3B,C).

These results indicated that neurons in the hippocampus (EC, CA3 and CA1) responded to noise bursts only in awake mice and almost not to tone bursts.

### Response Latencies and Thresholds of Neurons in the Hippocampus, MS and A1

**FIGURE 3:**
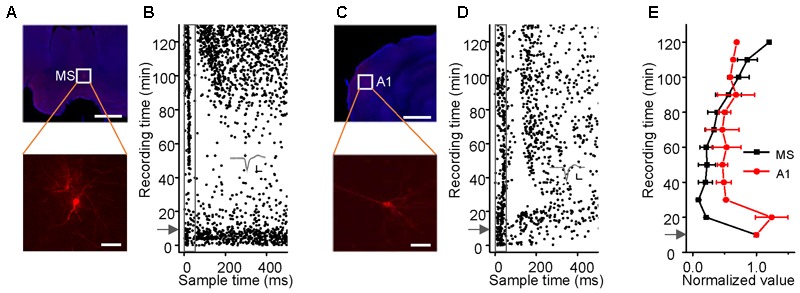
Effects of sodium pentobarbital on the responses of MS and A1 neurons to noise. **(A–D)** Noise-evoked responses (**B,D**, raster plots) of neurons (**A,C**, lower panels, Scale bar, 50 μm) located (**A,C**, upper panels, Scale bar, 1000 μm) in the MS and A1 before and after sodium pentobarbital injection (arrows). Inset: 20 randomly selected superimposed spike waveforms. Scale: 40 pA, 0.5 ms. **(E)** Changes in the spike rate (mean ± SEM) of a single neuron in the MS (black, *n* = 3) and A1 (red, *n* = 3) after the injection. Each point represents a spike rate normalized by that measured during a 10-min period prior to the injection.

The hippocampus has been thought to receive an indirect projection from the AC (Vinogradova, 1975; Steward, 1976; Germroth et al., 1989; Moxon et al., 1999; Winer and Schreiner, 2011) and a direct one from the MS (Vinogradova, 1975; Baisden et al., 1984; Kaifosh et al., 2013) for processing auditory information, such as the CS in auditory fear conditioning (Moita et al., 2003, 2004). We loosely patched neurons in the MS (**Figure 3A**) and A1 (**Figure 3C**) in awake mice and studied the effects of pentobarbital anesthesia (60–70 mg/kg, i.p.) on their responses to noise bursts and spontaneous discharges (**Figures 3B,D**). The evoked responses and spontaneous discharges of MS neurons faded over the 30 min after the injection and then recovered gradually over the 120 min after that (**Figures 3B,E**, *black lines*), as did those of the hippocampal neurons [repeated-measures ANOVA, *F*_(3,6)_ = 1.144, *P* = 0.404]. The responses of A1 neurons to 90 dB SPL noise bursts slightly decreased after pentobarbital injection, whereas their spontaneous discharges initially increased and then decreased (**Figures 3D,E**, *red line*). Thus, the effects of sodium pentobarbital on A1 were different from those on MS [repeated-measures ANOVA, *F*_(1,2)_ = 24.150, *P* = 0.039] and hippocampal neurons [repeated-measures ANOVA, *F*_(3,9)_ = 24.834, *P* = 0.013].

**FIGURE 4:**
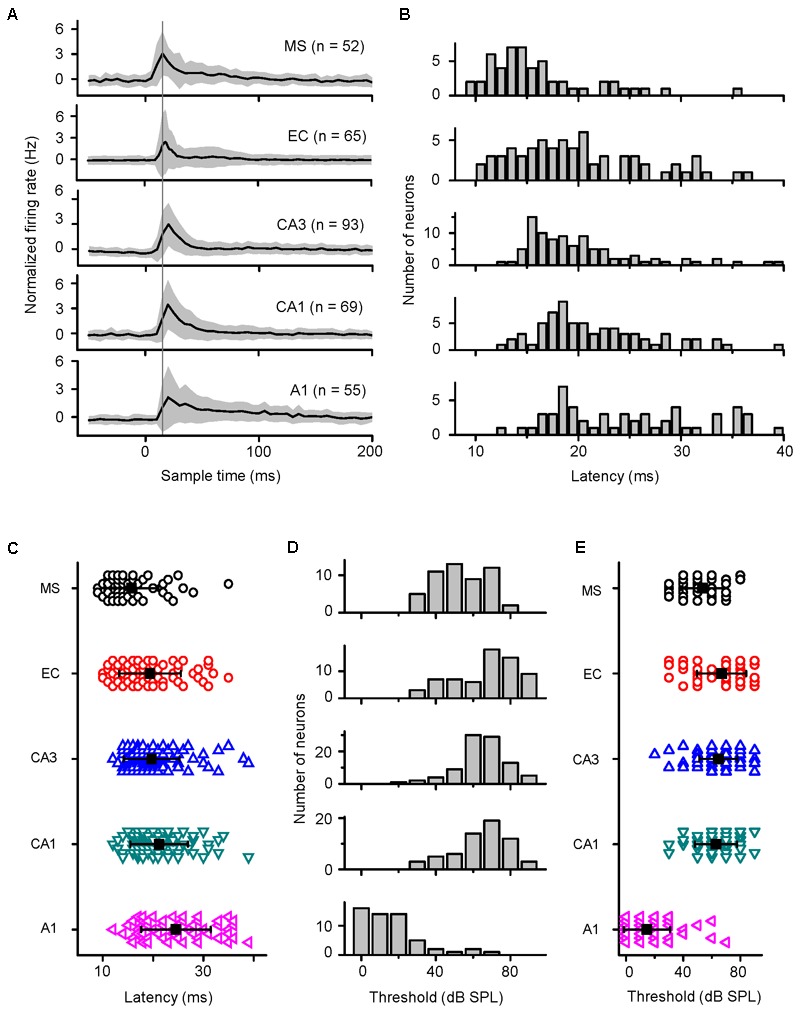
Differences in the response latencies and thresholds among the MS, EC, CA3, CA1, and A1 regions. **(A)** Normalized average response PSTHs computed for all acoustic response cells recorded in five brain areas. Shaded areas indicate SD. The vertical black line indicates the time of the peak responses in MS. **(B,D)** Distributions of response latencies (at 90 dB SPL) and thresholds of neurons in the MS, EC, CA3, CA1, and A1 regions of awake mice to noise. **(C,E)** Comparison of onset latencies **(B)** and thresholds **(D)** among the MS, EC, CA3, CA1, and A1 neurons. Bar represents mean ± SD.

In awake mice, the response latencies and thresholds of the neurons in the MS, EC, CA3, CA1, and A1 to noise bursts were studied. Of the 276 patched neurons in the MS, 52 neurons responded to noise bursts, showing a monotonic spike count-intensity function. Only 3/52 cells responded to a specific frequency only at high intensity (90 dB SPL), as did the hippocampal neurons. In A1, 26 of the 55 neurons responded only to noise bursts, whereas the remaining 29 neurons responded to both noise and tone bursts. Their TRFs were V-shaped. The normalized average response PSTHs are displayed in the MS, EC, CA3, CA1, and A1 (**Figure 4A**). We also measured the response latencies and thresholds of the neurons recorded in the MS, EC, CA3, CA1, and A1 with noise burst stimuli (**Figures 4B,D**). The distributions of the response latencies and thresholds were almost normal. We calculated the grand average latencies for the MS, EC, CA3, CA1, and A1 (15.731 ± 5.296, 19.385 ± 6.151, 19.709 ± 5.567, 21.217 ± 5.716, and 24.556 ± 6.914, respectively) (**Figure 4C**). The one-way ANOVA statistic indicated that the average latency for MS neurons was significantly shorter than that of the EC, CA3, CA1, or A1 neurons [*F*_(4,329)_ = 14.979, *P* < 0.001; Scheffe test, MS vs. EC: *P* = 0.028, MS vs. CA3: *P* = 0.005, MS vs. CA1: *P* < 0.001, and MS vs. A1: *P* < 0.001]. The average latencies for the EC, CA3, and CA regions were significantly shorter than was that of A1 (EC vs. A1: *P* < 0.001; CA3 vs. A1: *P* < 0.001; and CA1 vs. A1: *P* = 0.049), and the average latencies of the EC, CA3, and CA1 were not significantly different from each other (EC vs. CA3: *P* = 0.998, EC vs. CA1: *P* = 0.519, and CA3 vs. CA1: *P* = 0.626).

We also calculated the grand average thresholds for the MS, EC, CA3, CA1, and A1 (53.462 ± 13.987, 66.923 ± 17.223, 64.623 ± 13.397, 62.899 ± 14.763, and 14.636 ± 16.241, respectively) (**Figure 4E**). The average threshold for A1 was significantly lower than that for the MS, EC, CA3, and CA1 [one-way ANOVA, *F*_(4,329)_ = 123.444, *P* < 0.001; Scheffe test, MS vs. A1: *P* < 0.001; EC vs. A1: *P* < 0.001; CA3 vs. A1: *P* < 0.001; and CA1 vs. A1: *P* < 0.001]. The average threshold for the MS was significantly lower than those for the EC, CA3, and CA1 regions (MS vs. EC: *P* < 0.001; MS vs. CA3: *P* = 0.001; and MS vs. CA1: *P* = 0.022), and the average thresholds of the EC, CA3, and CA1 regions were not significantly different from each other (EC vs. CA3: *P* = 0.925; EC vs. CA1: *P* = 0.664; and CA3 vs. CA1: *P* = 0.971). Since the A1 latency was longer and the MS latency was shorter than the CA3 and CA1 latencies, it is most likely that auditory information sent to the hippocampus comes from the non-lemniscal pathway, not the lemniscal one.

### Effects of Inactivation of A1 or the MS on the Auditory Responses of the CA3 and EC Regions

**FIGURE 5:**
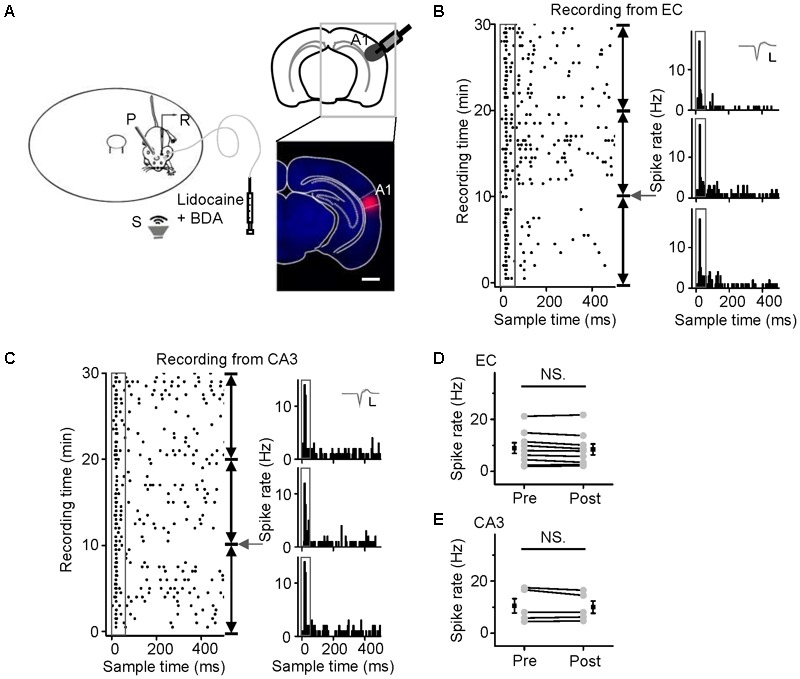
No effects of A1 inactivation on noise-evoked responses of neurons in the EC and CA3 regions were observed. **(A)** Diagram of the recording and lidocaine (co-applied with BDA) injection system in awake, head-fixed mice (left panel) and the location of inactivated A1 in a frontal section, indicated by BDA fluorescence (right panel). Scale bar, 1000 μm. **(B,C)** The responses to noise stimuli (80 dB SPL, 50 ms; gray boxes) of neurons in the EC **(B)** and CA3 regions **(C)** before (the lower panels, PSTHs) and after lidocaine application (arrows), as displayed by raster plots (left) and PSTHs (right). Each PSTH was collected every 10 min. Inset: 20 randomly selected superimposed spike waveforms. Scale: 40 pA, 0.5 ms. **(D,E)** Changes in the onset spike rates of EC **(D)** and CA3 **(E)** neurons after inactivation of A1 with lidocaine. NS, not significant. Error bars represent SEM.

To examine whether auditory information sent to the hippocampus comes from the MS or A1, i.e., from the non-lemniscal or lemniscal pathway, respectively, we injected lidocaine (a voltage-gated sodium channel blocker) into the ipsilateral A1 or the MS via an implanted cannula, while loose-patch recordings were performed from neurons in the EC and CA3 regions (**Figure 5A**, *left panel*). The injection sites were confirmed by the fluorescence of co-applied BDA mixed with lidocaine (**Figure 5A**, *right panel*). As shown in **Figure 5**, after the inactivation of A1, the auditory responses and spontaneous discharges of the EC and CA3 neurons 10 min after a lidocaine injection (**Figures 5B,C**, *middle panels*, PSTHs) were almost the same as those recorded before that (**Figures 5B,C**, *lower panels*, PSTHs). The pooled data from nine EC neurons (**Figure 5D**) and five CA3 neurons (**Figure 5E**) showed no difference in discharges between the 10 min before and the 10 min after the inactivation of A1 (paired *t*-test, *t* = 1.798, df = 8, *P* = 0.110 for EC; *t* = 0.336, df = 4, *P* = 0.754 for CA3). Thus, the hippocampal auditory inputs did not come from A1.

**FIGURE 6:**
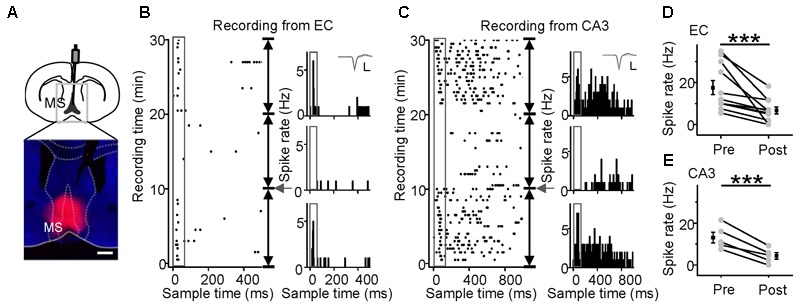
Effects of MS inactivation on the responses of EC and CA3 neurons to noise. MS inactivation (**A**, Scale bar, 500 μm) reduced the responses of neurons in the EC **(B)** and CA3 **(C)** regions of awake mice to noise stimuli (50 ms, 80 dB SPL). The responses are displayed by the raster plots (left) and PSTHs (right). Inset: 20 randomly selected superimposed spike waveforms. Scale: 40 pA, 0.5 ms. **(D,E)** Changes in onset spike rates of EC **(D)** and CA3 **(E)** neurons after inactivation of MS with lidocaine. ^∗∗∗^*P* < 0.001. Error bars represent SEM.

Medial septal neurons directly project to the EC and evoke hippocampal responses (Vinogradova, 1975; Kaifosh et al., 2013). Therefore, we applied lidocaine to the MS (**Figure 6A**). The raster plot showed that the noise-evoked responses and spontaneous discharges of both EC (**Figure 6B**) and CA3 (**Figure 6C**) neurons could be completely inhibited by lidocaine. All recorded neurons in the EC (**Figure 6D**, paired *t*-test, *t* = 6.436, df = 10, *P* < 0.001) and CA3 (**Figure 6E**, paired *t*-test *t* = 7.865, df = 4, *P* < 0.001) regions were significantly silenced. Therefore, the hippocampal auditory inputs came from the MS.

### Behavioral Responses to Auditory Fear Conditioning in Awake and Anesthetized Mice

**FIGURE 7:**
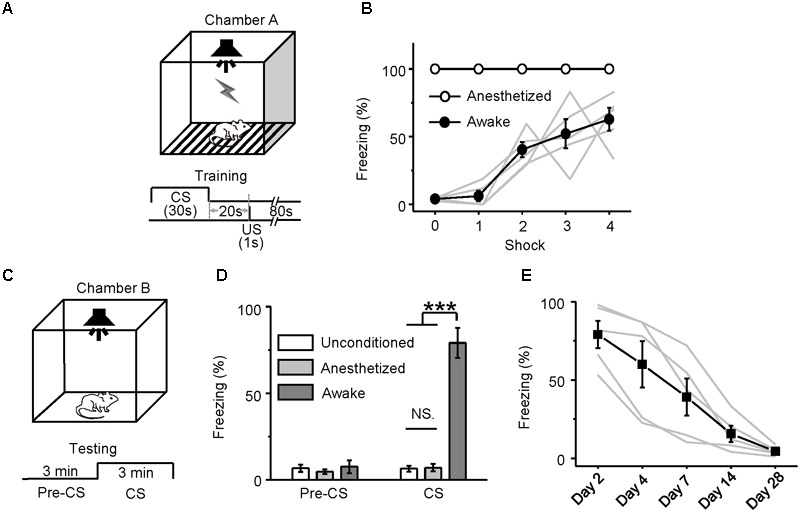
Behavioral responses of auditory fear conditioning in awake and anesthetized mice. **(A)** Training Chamber A and the training paradigm. **(B)** Freezing responses (mean ± SEM) of awake (dots; *n* = 5, gray lines: individual) and anesthetized (circles; *n* = 5) mice to auditory fear conditioning (four trials). CS, 30-s noise burst at 80 dB SPL; US, 1-s footshock delivered 20 s after CS. **(C)** Testing Chamber B and the testing paradigm. The testing included a 3-min adaptation (pre-CS) and a 3-min CS (noise burst at 80 dB SPL). **(D)** Freezing responses (mean ± SEM) among awake-unconditioned (white), anesthetized-conditioned (gray) and awake-conditioned (black) groups of mice observed during the pre-CS and CS presentations 24 h after the training were compared with each other. **(E)** Freezing responses of the awake-conditioned mice (*n* = 5, black: average, gray: individual) tested over the 28 days after the training. ^∗∗∗^*P* < 0.001. NS, not significant.

Neurons in the MS (Kaifosh et al., 2013) and hippocampus (O’Keefe and Nadel, 1978; Sacchetti et al., 1999; Moita et al., 2003; Maren and Holt, 2004; Czerniawski et al., 2012; Robinson and Bucci, 2012; Donzis et al., 2013; Kaifosh et al., 2013) are involved in auditory fear conditioning. Our *in vivo* loose-patch recordings indicated that neurons in the MS, EC, CA3, and CA1 regions responded to noise burst stimuli only in awake mice and not in pentobarbital-anesthetized mice. That is, the neurons in the MS, EC, CA3, and CA1 were silenced under pentobarbital anesthesia. This finding raised a question of whether auditory fear conditioning could occur in sodium pentobarbital-anesthetized animals even though they could not show conditioned behavioral responses. Fifteen mice were equally divided into three subgroups: awake-unconditioned, anesthetized-conditioned and awake-conditioned. Since the MS and hippocampal neurons responded well to noise burst stimuli, noise bursts might be the best CS. Therefore, mice were fear-conditioned with a 30-s-long noise burst (80 dB SPL), followed by a footshock with a 20-s gap (**Figure 7A**).

During the conditioning trials, the freezing response of the awake mice gradually increased from the first to the fourth trial (**Figure 7B**, *filled circles*), whereas the anesthetized mice (**Figure 7B**, *open circles*) clearly did not show any movement. On the following day, they were tested for freezing responses by presenting 3 min of CS compared to 3 min of pre-sound in a different context (**Figure 7C**). The awake mice showed freezing responses at a much higher percentage (79.07 ± 8.64%) than did the anesthetized mice [7.09 ± 2.13%, one-way ANOVA, *F*_(2,12)_ = 63.628, *P* < 0.001; Scheffe test, awake vs. anesthetized: *P* < 0.001] and the awake-unconditioned mice (6.51 ± 1.71%, awake vs. unconditioned: *P* < 0.001) (**Figure 7D**). Auditory fear conditioning was acquired only by the awake mice. The retention of the acquired freezing response was tested 2, 4, 7, 14, and 28 days after the training (**Figure 7E**). The auditory fear memory almost disappeared on day 14, which was consistent with the findings of a previous study (Gold et al., 1985).

### Effect of MS Inactivation on the Acquisition of Auditory Fear Conditioning

Our electrophysiological and pharmacological experiments showed that the MS provided auditory input to the hippocampus, that anesthesia silenced the neurons in the MS and the hippocampus, and that auditory fear conditioning could not be acquired by anesthetized mice. Whether MS inactivation could block the acquisition of auditory fear conditioning remains a question. Neurons in the MS and the hippocampus both strongly responded to noise bursts rather than to tone bursts. If the septo-hippocampal pathway is a route that specifically carries the noise bursts information, then MS inactivation would block the behavioral response trained with noise bursts but not those trained with tone bursts. Therefore, we studied whether the behavioral responses to noise bursts (80 dB SPL) and tone bursts (80 dB SPL) were affected by MS inactivation.

**FIGURE 8:**
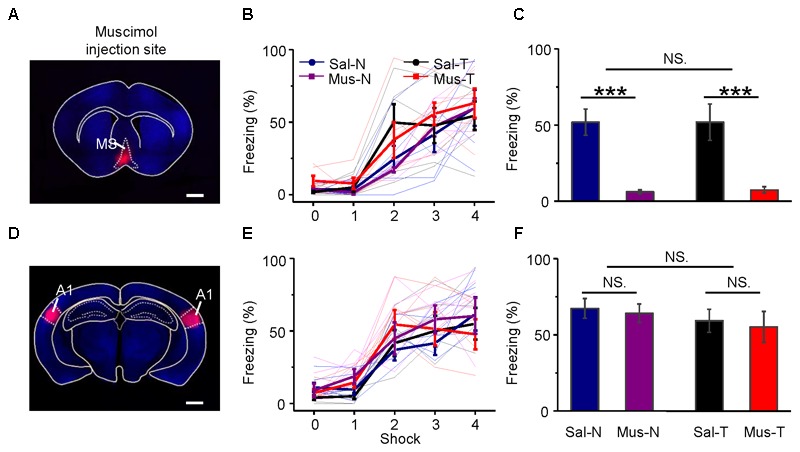
Effects of inactivation of the MS or A1 on conditioning and retention. Injection of muscimol in the MS **(A)** had no effect on learning **(B)**, but it abolished retention **(C)**. On the other hand, that of A1 **(D)** had no effect on them **(E,F)**. **(A,D)** Frontal sections across the MS and A1, respectively. Scale bar, 1000 μm. **(B,E)** Auditory fear conditioning learning curves. **(C,F)** Differences in retention of conditioned behavior, namely, freezing, between inactivated and saline-injected mice. Each bar in **(C,F)** indicates mean ± SEM. Each data point is based on six mice. Mus., muscimol; Sal., saline; N, conditioning noise burst; T, conditioning tone burst. Sal-N, blue; Sal-T, black; Mus-N, purple; Mus-T, red; NS, not significant; ^∗∗∗^*P* < 0.001.

The MS was inactivated by an injection of muscimol (an agonist of GABA_A_ receptors). Its effect lasts more than 1 h (Martin and Ghez, 1999), and the half-life time of its effect is longer than that of lidocaine (Martin and Ghez, 1999). Therefore, the inactivation of the MS with muscimol lasts long enough for the long-lasting auditory fear training. Twenty-four mice were equally divided into four groups. Muscimol (Mus) or saline (Sal, as the control) was injected into the MS of each mouse via an implanted cannula 10–15 min before training. Then, each mouse was trained with noise bursts (N) or tone bursts of 2.5 kHz (T). The four groups were Mus-N, Mus-T, Sal-N, and Sal-T. The injection site was confirmed by the fluorescence of the co-applied BDA into the MS (**Figure 8A**). During acquisition training, all groups of mice showed increased freezing (**Figure 8B**), but there were no differences among them [repeated-measures ANOVA, *F*_(3,15)_ = 0.838, *P* = 0.494]. In the retention testing 24 h after the conditioning, however, the average freezing response of the Mus-N (6.17 ± 1.28) and Mus-T (7.41 ± 2.09) mice was significantly lower than that of the Sal-N (51.89 ± 8.65) and Sal-T mice [51.92 ± 11.91, one-way ANOVA, *F*_(3,20)_ = 18.374, *P* < 0.001, Scheffe test: Sal-N vs. Mus-N, *P* < 0.001; and Sal-T vs. Mus-T, *P* < 0.001], whereas the freezing responses between the Mus-N and Mus-T and the Sal-N and Sal-T mice were not different (Sal-N vs. Sal-T, *P* = 1.000; Mus-N vs. Mus-T, *P* = 0.999) (**Figure 8C**).

Although A1 is not required for the conditioning of fear responses to simple acoustic stimuli (LeDoux et al., 1984; Romanski and LeDoux, 1992a,b; Armony et al., 1997), we bilaterally applied muscimol or saline to A1 (**Figure 8D**) as a control to the above experiment. To confirm that our infusion drugs were targeting A1 specifically, we estimated the anatomical spread of muscimol by inspecting cannula tracks and using the fluorescence of the co-applied BDA mixed with muscimol. The histological analysis revealed cannula hits within A1 (Supplementary Figure S4) and the fluorescence of the co-applied BDA were mainly in A1 (**Figure 8D**). The freezing responses to the noise CS during acquisition training [**Figure 8E**, repeated-measures ANOVA, *F*_(3,15)_ = 0.544, *P* = 0.659] and retention testing [**Figure 8F**, one-way ANOVA, *F*_(3,20)_ = 0.474, *P* = 0.704, Scheffe test: Sal-N vs. Mus-N, *P* = 0.993; and Sal-T vs. Mus-T, *P* = 0.988] were not different from those to the tone CS in either the muscimol-treated (Mus-N vs. Mus-T, *P* = 0.883) or the saline-treated (Sal-N vs. Sal-T, *P* = 0.906) mice.

These results indicated that MS inactivation induced mice amnesia, but A1 inactivation did not. Unexpectedly, MS inactivation blocked not only the noise-induced freezing responses but also the tone-induced responses.

### Auditory Fear Conditioning Independent of the Frequency of Conditioned Sound

**FIGURE 9:**
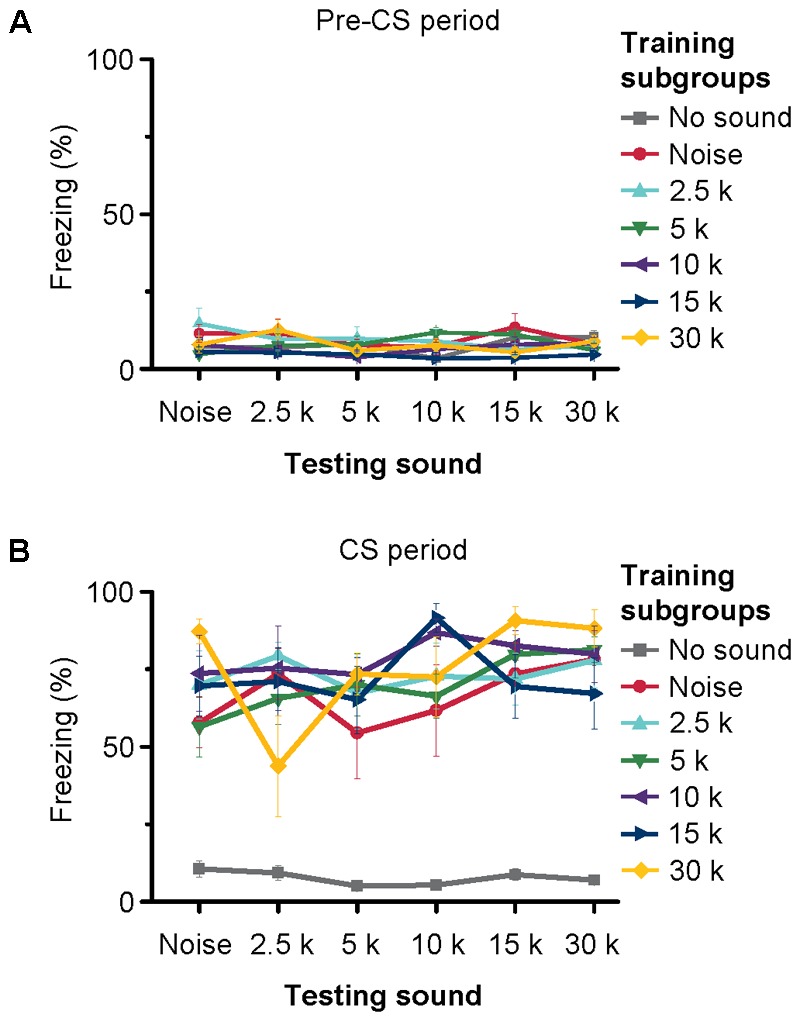
Freezing of mice trained and tested with different sounds. The seven separate lines indicate the average freezing% of pre-CS period **(A)** and CS-period **(B)** of seven different training subgroups to different CSs. Each training subgroup contains six samples, depicted are the mean freezing ± SEM %.

We trained the animals with noise bursts and tone bursts of 2.5 kHz. MS inactivation blocked both the noise- and tone-conditioned fear memory (**Figure 8C**). This result indicated that both the noise and tone might via MS to induce behavioral response. Our electrophysiology results showed that most of MS neurons responded to noise bursts, and a small number of neurons also responded to tone stimulus but they did not show any frequency preference, as did those in hippocampus. This implied that both the noise- and tone-bursts could induce the same behavioral response in the auditory fear conditioning. To confirm this possibility, we devised another behavioral experiment. In this experiment, each mouse was conditioned to one kind of auditory stimulus but was tested with multiple sounds 24 h after the training (Supplementary Figure S5). Forty-two mice were equally divided into seven subgroups for training with either no-sound, noise, 2.5-, 5-, 10-, 15-, or 30-kHz tones (**Figure 9**). Each mouse was tested for the freezing response to the sounds (noise, 2.5-, 5-, 10-, 15-, and 30-kHz tones) 24 h after the training. The interval between two testing sessions was 1 h. The order of the different testing sounds was contained in a randomized sequence. Each testing included 3 min without sound (pre-CS testing) and 3 min with sound (CS testing). The freezing patterns during pre-CS (**Figure 9A**) and CS (**Figure 9B**) testing with different sounds for each subgroup were averaged. The results were analyzed with a three-way analysis of covariance using training, testing and testing order as factors and pre-CS as the covariate. The freezing patterns for all pre-CS were not significantly different [*F*_(1,128)_ = 0.051, *P* = 0.822]. The evaluation mixed the training, testing and testing order as independent variables with the data for pre-CS testing because the covariance was significantly different [*F*_(123,128)_ = 3.452, *P* < 0.001]. However, there were no significant differences in testing with different sounds [*F*_(5,128)_ = 0.823, *P* = 0.535] or in the sound-testing order [*F*_(5,128)_ = 1.604, *P* = 0.164]. The interactions between training and testing [*F*_(30,128)_ = 1.176, *P* = 0.263], training and testing order [*F*_(30,128)_ = 0.685, *P* = 0.885], and testing and testing order [*F*_(24,128)_ = 0.982, *P* = 0.494] and among training, testing and testing order [*F*_(22,128)_ = 1.421, *P* = 0.116] were not significant. The only difference was in the training groups [*F*_(6,128)_ = 31.842, *P* < 0.001]. *Post hoc* analysis (Student–Newman–Keuls test) separated the two subsets at the *P* = 0.05 level. The training without the sound subgroup against the training with different sounds was the subset with *P* < 0.05. The other subset was the comparisons among the subgroups trained with different sounds, in which there was no significant difference (Student–Newman–Keuls test, *P* = 0.160). These results indicated that the freezing response induced by auditory fear conditioning was independent of the frequency of the sound used as the CS.

## Discussion

In the current studies, we explored the auditory response properties of hippocampal neurons, the auditory pathway to the hippocampus, and the role of hippocampal auditory responses in auditory fear conditioning using electrophysiological and behavioral assays combined with pharmacological manipulations. We made four major findings. (1) Neurons in the hippocampus (EC, CA3, and CA1 regions) responded only to noise burst stimuli in awake mice. (2) Hippocampal auditory inputs came from the MS and not from A1. (3) MS inactivation impaired the acquisition of auditory fear conditioning. (4) Auditory fear conditioning was not associated with a specific frequency of sound. These findings provided the following implications.

The hippocampus is a well-known structure of the limbic system. In our current study, approximately 15% of the neurons in the hippocampus responded to noise burst stimuli (**Figures 1B–D, 2, 4, 5, 6B,C**). This result was consistent with those of the previous studies demonstrating that neurons in the limbic system, such as in the amygdala, striatum, cingulate cortex and septum, can be driven more effectively by noise bursts and clicks (Vinogradova, 1975; Bordi and LeDoux, 1992; Chen et al., 2014). A few neurons in the hippocampus responded to intense tone bursts (**Figures 2B,C**), as reported previously (Kaifosh et al., 2013; Chen et al., 2014). However, no clear TRF or frequency preferences were observed (**Figure 2B**), unlike the neurons in the amygdala and the striatum, which are tonotopically organized (Bordi and LeDoux, 1992; Chen et al., 2012). Therefore, the auditory pathway to the hippocampus is different from that to the amygdala and the striatum.

The auditory system includes the lemniscal and non-lemniscal ascending pathways (Hu et al., 1994; Jones, 2003). The non-lemniscal pathway, or the limbic system, is sensitive to temperature and drugs, especially to anesthetics (Vinogradova, 1975; Dickinson et al., 2003; Kaifosh et al., 2013). This is why the noise-evoked responses were blocked by sodium pentobarbital (**Figure 1**). Therefore, auditory sub-threshold responses have been recorded only in awake subjects (Abe et al., 2014). However, several studies have recorded auditory evoked potentials and single units in anesthetized animals (Miller and Freedman, 1995; Krause et al., 2003; Dissanayake et al., 2008). This may have been due to the differences in extracellular recording and/or anesthetics.

Although the non-lemniscal pathway from the brainstem reticular formation relayed by the MS projects to the hippocampus (Vinogradova, 1975; Baisden et al., 1984; Moxon et al., 1999; Kaifosh et al., 2013), auditory information from the lemniscal pathway is thought to be processed by the association cortex prior to entering the hippocampus via the EC (Vinogradova, 1975; Steward, 1976; Germroth et al., 1989; Moxon et al., 1999). However, the responses of hippocampal neurons were different from those of lemniscal pathway neurons. Additionally, the shorter latency than the A1 neurons (**Figures 4A,B**) and previous reports (Bickford-Wimer et al., 1990; Moxon et al., 1999) did not support this processing. Nevertheless, the onset latency of the MS neurons was shorter than those of the EC, CA3, and CA1 neurons (**Figures 4A,B**). It seems that the auditory inputs to the hippocampus come from the MS rather than from A1. The direct evidence is that the sound-evoked discharges in the EC and CA3 neurons were blocked by silencing the MS (**Figure 6**) rather than by silencing A1 (**Figure 5**). In addition, this auditory information processing pathway from the MS to the hippocampus has been doubly confirmed by behavioral measurements of auditory fear conditioning of silenced MS animal controls to silenced A1; inactivation of MS but not of A1 blocked the CS-induced fear behavior (**Figure 8**). This septo-hippocampal pathway also processes other sensory information, e.g., visual, olfactory and somatosensory information (Vinogradova, 1975; O’Keefe and Nadel, 1978; Kaifosh et al., 2013).

Auditory fear conditioning includes trace fear conditioning and delay fear conditioning. Trace fear conditioning has a temporal gap between the tone (CS) and aversive electrical footshock (US), whereas delay fear conditioning does not. It is well known that the hippocampus is required for trace fear conditioning but is not required for delay fear conditioning (Raybuck and Lattal, 2011). Pentobarbital anesthesia can inactivate the neurons in the hippocampus (**Figure 1**) and the MS (**Figure 3B**). Therefore, we could not observe the acquired behavioral responses of auditory fear conditioning in anesthetized mice (**Figure 7D**). This finding was consistent with those of previous papers (Weinberger et al., 1984; Ghoneim et al., 1992), although few studies have reported that auditory fear conditioning occurs in anesthetized animals (Edeline and Neuenschwander-El, 1988; Pang et al., 1996). However, sodium pentobarbital injected intraperitoneally did not only inactivated the neurons in the hippocampus and the MS but also many other neurons. To provide special evidence for the hippocampal auditory pathway involvement in auditory fear conditioning, we silenced A1 and the MS. Inactivation of MS neurons blocked the acquisition of auditory fear conditioning memory (**Figures 8A–C**). This result was similar to that of a previous study that found that muscimol infused into the MS impairs long-term memory but not short-term memory in inhibitory avoidance and the water maze place-learning task and enhanced alternation tasks (Nagahara and McGaugh, 1992). Although a previous study assumed that the MS is not required for auditory-cue fear conditioning (Calandreau et al., 2007), possible explanations for the discrepancy between this previous study and our findings are the several technical and methodological differences. The previous study used lidocaine to inactivate the MS, the effects of which last approximately 10–15 min; in this study, we used muscimol to inactivate the MS and thereby block the acquisition of auditory fear conditioning completely. It is well known that the hippocampus receives abundant inputs from the MS, and a previous study reported that inactivation of the hippocampus disrupted auditory trace fear conditioning (Maren and Holt, 2004; Raybuck and Lattal, 2011). Thus we have enough evidence to speculate that the MS probably plays a critical role in auditory fear conditioning.

Although inactivation of A1 has not been shown to block auditory fear conditioning memory (**Figures 8D–F**), congruent with the findings of previous studies (LeDoux et al., 1984; Romanski and LeDoux, 1992a,b; Armony et al., 1997), we cannot completely exclude the possibility that under certain learning paradigms, some functional connections between the AC and the hippocampus can be established through the mechanism of plasticity since weak projections from the AC to the hippocampus do exist (Tranel et al., 1988).

Auditory fear conditioning has been thought to associate an auditory pathway with behavior. The auditory pathway from the brainstem reticular formation relayed by the MS projecting to the hippocampus is non-lemniscal (Vinogradova, 1975; Baisden et al., 1984; Moxon et al., 1999). The non-lemniscal neurons innervated by reticular projections of lemniscal neurons lose the frequency specificity of sound (Aitkin and Webster, 1972; Phillips and Irvine, 1979) and are driven effectively by noise (Vinogradova, 1975; Chen et al., 2012, 2014). Neurons in the MS strongly responded to noise bursts rather than to tone bursts. If the septo-hippocampal pathway is a specifically transmits the noise bursts information, then MS inactivation would only block the behavioral response trained with noise bursts. Unexpectedly, MS inactivation blocked both the noise- and tone-conditioned fear memory (**Figure 8C**). This result indicated that both the noise and tone might via MS to induce the same behavioral response. To confirm this possibility, we trained the animals with one kind of auditory stimulus but tested them with multiple sounds (Supplementary Figure S5). No significant differences were observed among the different conditioned sounds (**Figure 9**). However, recently, a study indicated that silencing the MS during conditioning specifically reduced noise-conditioned (Zhang et al., 2018). This was not consistent with our results. Our results showed that the inactivation of the MS during conditioning blocked both the noise- and tone-conditioned fear responses (**Figure 8C**). The explanation for the discrepancy may be due to the different behavioral training procedures; we used trace fear conditioning model in this study, whereas they adopted delay fear conditioning. Another reason is that the MS inactivation may have disrupted fear conditioning through other mechanisms. For example, it is well known that MS inactivation impairs theta rhythms in the hippocampus, which may, in turn, impair fear conditioning. Additionally, the septo-hippocampal pathway also processes other sensory information, e.g., visual, olfactory and somatosensory information. The auditory fear conditioning behavior was independent of the frequency characteristics of the conditioned sound (**Figure 9**). This was consistent with previous observation that different frequencies of conditioned stimuli could elicit freezing responses (Laxmi et al., 2003). Additional, our results were also supported by the other literature. For example, the animal acquired fear of the CS+ (10-kHz) and generalized to the CS- (2-kHz) after auditory fear conditioning training (Antunes and Moita, 2010). A recent study showed that conditioning with different auditory CSs recruit distinct forms of amygdaloid nucleus synaptic plasticity, but the freezing responses showed no significant differences among the three different auditory CSs groups (Park et al., 2016). Our results may be in contradiction with studies about discriminative fear conditioning. The different experiment conditions may account for this divergence. Therefore, the training associates the auditory non-lemniscal pathway with behavior in the acquisition of auditory fear conditioning memory. However, what is the role of noise responses in the hippocampus? The noise responses in the hippocampus have identified a population of neurons that can be associated with behavior. The different thresholds of this population of neurons would indicate that the neurons could be activated both with difficulty and easily. Thus, in auditory fear conditioning, the noise responses of the hippocampus demonstrated that the number of neurons that can be involved is associated with behavior during training. The auditory non-lemniscal neurons are involved in multisensory integration (Calford and Webster, 1981; Winer and Morest, 1983). Therefore, this hippocampal auditory pathway may provide the basis for the processing of multiple types of sensory information in the limbic system.

This study, we have provided functional evidences that the hippocampus, as relayed by the MS, only responds to noise stimuli in awake subjects, and the noise responses of hippocampal neurons have identified a population of neurons that can be associated with behavior, which is independent of the characteristic of conditioned sound.

## Author Contributions

ZX designed the research and wrote the paper. CX, YL, JX, and XG performed the research and analyzed the data. All authors read and approved the manuscript.

## Conflict of Interest Statement

The authors declare that the research was conducted in the absence of any commercial or financial relationships that could be construed as a potential conflict of interest.

## References

[B1] Abe R. Sakaguchi T. Matsumoto N. Matsuki N. Ikegaya Y. (2014). Sound-induced hyperpolarization of hippocampal neurons. *Neuroreport* 25 1013–1017. 10.1097/WNR.0000000000000206 25050474

[B2] Aitkin L. M. Webster W. R. (1972). Medial geniculate body of the cat: organization and responses to tonal stimuli of neurons in ventral division. *J. Neurophysiol.* 35 365–380. 10.1152/jn.1972.35.3.365 5029955

[B3] Antunes R. Moita M. A. (2010). Discriminative auditory fear learning requires both tuned and nontuned auditory pathways to the amygdala. *J. Neurosci.* 30 9782–9787. 10.1523/jneurosci.1037-10.2010 20660260 PMC6632832

[B4] Armony J. L. Servan-Schreiber D. Romanski L. M. Cohen J. D. LeDoux J. E. (1997). Stimulus generalization of fear responses: effects of auditory cortex lesions in a computational model and in rats. *Cereb. Cortex* 7 157–165. 10.1093/cercor/7.2.157 9087823

[B5] Baisden R. H. Woodruff M. L. Hoover D. B. (1984). Cholinergic and non-cholinergic septo-hippocampal projections: a double-label horseradish peroxidase-acetylcholinesterase study in the rabbit. *Brain Res.* 290 146–151. 10.1016/0006-8993(84)90745-5 6692131

[B6] Bickford-Wimer P. C. Nagamoto H. Johnson R. Adler L. E. Egan M. Rose G. M. (1990). Auditory sensory gating in hippocampal neurons: a model system in the rat. *Biol. Psychiatry* 27 183–192. 10.1016/0006-3223(90)90648-L 2294981

[B7] Bordi F. LeDoux J. (1992). Sensory tuning beyond the sensory system: an initial analysis of auditory response properties of neurons in the lateral amygdaloid nucleus and overlying areas of the striatum. *J. Neurosci.* 12 2493–2503. 10.1523/JNEUROSCI.12-07-02493.1992 1613543 PMC6575825

[B8] Bouton M. E. Bolles R. C. (1980). Conditioned fear assessed by freezing and by the suppression of three different baselines. *Anim. Learn. Behav.* 8 429–434. 10.3758/BF03199629

[B9] Brankack J. Buzsaki G. (1986). Hippocampal responses evoked by tooth pulp and acoustic stimulation: depth profiles and effect of behavior. *Brain Res.* 378 303–314. 10.1016/0006-8993(86)90933-9 3730880

[B10] Buccafusco J. J. (2009). *Methods of Behavior Analysis in Neuroscience.* Boca Raton, FL: CRC Press. 21204335

[B11] Calandreau L. Jaffard R. Desmedt A. (2007). Dissociated roles for the lateral and medial septum in elemental and contextual fear conditioning. *Learn. Mem.* 14 422–429. 10.1101/lm.531407 17554087 PMC1896092

[B12] Calford M. B. Webster W. R. (1981). Auditory representation within principal division of cat medial geniculate body: an electrophysiology study. *J. Neurophysiol.* 45 1013–1028. 10.1152/jn.1981.45.6.1013 7252528

[B13] Chen G. D. Manohar S. Salvi R. (2012). Amygdala hyperactivity and tonotopic shift after salicylate exposure. *Brain Res.* 1485 63–76. 10.1016/j.brainres.2012.03.016 22464181 PMC5319430

[B14] Chen G. D. Radziwon K. E. Kashanian N. Manohar S. Salvi R. (2014). Salicylate-induced auditory perceptual disorders and plastic changes in nonclassical auditory centers in rats. *Neural Plast.* 2014:658741. 10.1155/2014/658741 24891959 PMC4033555

[B15] Czerniawski J. Ree F. Chia C. Otto T. (2012). Dorsal versus ventral hippocampal contributions to trace and contextual conditioning: differential effects of regionally selective NMDA receptor antagonism on acquisition and expression. *Hippocampus* 22 1528–1539. 10.1002/hipo.20992 22180082

[B16] Dickinson R. Awaiz S. Whittington M. A. Lieb W. R. Franks N. P. (2003). The effects of general anaesthetics on carbachol-evoked gamma oscillations in the rat hippocampus in vitro. *Neuropharmacology* 44 864–872. 10.1016/S0028-3908(03)00083-2 12726818

[B17] Dissanayake D. W. Zachariou M. Marsden C. A. Mason R. (2008). Auditory gating in rat hippocampus and medial prefrontal cortex: effect of the cannabinoid agonist WIN55,212-2. *Neuropharmacology* 55 1397–1404. 10.1016/j.neuropharm.2008.08.039 18809420

[B18] Donzis E. J. Rennaker R. L. Thompson L. T. (2013). Fear conditioning alters neuron-specific hippocampal place field stability via the basolateral amygdala. *Brain Res.* 1525 16–25. 10.1016/j.brainres.2013.06.015 23791951

[B19] Edeline J. M. Neuenschwander-El M. N. (1988). Retention of CS-US association learned under ketamine anesthesia. *Brain Res.* 457 274–280. 10.1016/0006-8993(88)90696-8 3219555

[B20] Fanselow M. S. (1980). Conditioned and unconditional components of post-shock freezing. *Pavlov. J. Biol. Sci.* 15 177–182. 7208128 10.1007/BF03001163

[B21] Germroth P. Schwerdtfeger W. K. Buhl E. H. (1989). Morphology of identified entorhinal neurons projecting to the hippocampus. A light microscopical study combining retrograde tracing and intracellular injection. *Neuroscience* 30 683–691. 10.1016/0306-4522(89)90161-9 2771045

[B22] Ghoneim M. M. Block R. I. Fowles D. C. (1992). No evidence of classical conditioning of electrodermal responses during anesthesia. *Anesthesiology* 76 682–688. 10.1097/00000542-199205000-00004 1575334

[B23] Gold P. E. Weinberger N. M. Sternberg D. B. (1985). Epinephrine-induced learning under anesthesia: retention performance at several training-testing intervals. *Behav. Neurosci.* 99 1019–1022. 10.1037/0735-7044.99.5.1019 3843300

[B24] Hu B. Senatorov V. Mooney D. (1994). Lemniscal and non-lemniscal synaptic transmission in rat auditory thalamus. *J. Physiol.* 479(Pt 2) 217–231. 10.1113/jphysiol.1994.sp020290 7799222 PMC1155741

[B25] Huang B. Liang F. Zhong L. Lin M. Yang J. Yan L. (2015). Latency of auditory evoked potential monitoring the effects of general anesthetics on nerve fibers and synapses. *Sci. Rep.* 5:12730. 10.1038/srep12730 26246365 PMC4526847

[B26] Itskov P. M. Vinnik E. Honey C. Schnupp J. Diamond M. E. (2012). Sound sensitivity of neurons in rat hippocampus during performance of a sound-guided task. *J. Neurophysiol.* 107 1822–1834. 10.1152/jn.00404.2011 22219030 PMC3331670

[B27] Jones E. G. (2003). Chemically defined parallel pathways in the monkey auditory system. *Ann. N. Y. Acad. Sci.* 999 218–233. 10.1196/annals.1284.033 14681146

[B28] Kaifosh P. Lovett-Barron M. Turi G. F. Reardon T. R. Losonczy A. (2013). Septo-hippocampal GABAergic signaling across multiple modalities in awake mice. *Nat. Neurosci.* 16 1182–1184. 10.1038/nn.3482 23912949

[B29] Krause M. Hoffmann W. E. Hajós M. (2003). Auditory sensory gating in hippocampus and reticular thalamic neurons in anesthetized rats. *Biol. Psychiatry* 53 244–253. 10.1016/s0006-3223(02)01463-4 12559658

[B30] Laxmi T. R. Stork O. Pape H. C. (2003). Generalisation of conditioned fear and its behavioural expression in mice. *Behav. Brain Res.* 145 89–98. 10.1016/S0166-4328(03)00101-3 14529808

[B31] LeDoux J. E. (2000). Emotion circuits in the brain. *Annu. Rev. Neurosci.* 23 155–184. 10.1146/annurev.neuro.23.1.155 10845062

[B32] LeDoux J. E. Sakaguchi A. Reis D. J. (1984). Subcortical efferent projections of the medial geniculate nucleus mediate emotional responses conditioned to acoustic stimuli. *J. Neurosci.* 4 683–698. 10.1523/JNEUROSCI.04-03-00683.1984 6707732 PMC6564820

[B33] Lein E. S. Hawrylycz M. J. Ao N. Ayres M. Bensinger A. Bernard A. (2007). Genome-wide atlas of gene expression in the adult mouse brain. *Nature* 445 168–176. 10.1038/nature05453 17151600

[B34] Maren S. Holt W. G. (2004). Hippocampus and Pavlovian fear conditioning in rats: muscimol infusions into the ventral, but not dorsal, hippocampus impair the acquisition of conditional freezing to an auditory conditional stimulus. *Behav. Neurosci.* 118 97–110. 10.1037/0735-7044.118.1.97 14979786

[B35] Martin J. H. Ghez C. (1999). Pharmacological inactivation in the analysis of the central control of movement. *J. Neurosci. Methods* 86 145–159. 10.1016/S0165-0270(98)00163-0 10065983

[B36] McHugh S. B. Marques-Smith A. Li J. Rawlins J. N. Lowry J. Conway M. (2013). Hemodynamic responses in amygdala and hippocampus distinguish between aversive and neutral cues during Pavlovian fear conditioning in behaving rats. *Eur. J. Neurosci.* 37 498–507. 10.1111/ejn.12057 23173719 PMC3638322

[B37] Mercer L. F. Jr. Remley N. R. (1979). Mapping of sensory-responsive cells in the septal area of the rat. *Brain Res. Bull.* 4 483–490. 10.1016/0361-9230(79)90032-7 487202

[B38] Miller C. L. Freedman R. (1995). The activity of hippocampal interneurons and pyramidal cells during the response of the hippocampus to repeated auditory stimuli. *Neuroscience* 69 371–381. 10.1016/0306-4522(95)00249-I 8552235

[B39] Moita M. A. Rosis S. Zhou Y. LeDoux J. E. Blair H. T. (2003). Hippocampal place cells acquire location-specific responses to the conditioned stimulus during auditory fear conditioning. *Neuron* 37 485–497. 10.1016/S0896-6273(03)00033-3 12575955

[B40] Moita M. A. Rosis S. Zhou Y. LeDoux J. E. Blair H. T. (2004). Putting fear in its place: remapping of hippocampal place cells during fear conditioning. *J. Neurosci.* 24 7015–7023. 10.1523/JNEUROSCI.5492-03.2004 15295037 PMC6729593

[B41] Moxon K. A. Gerhardt G. A. Bickford P. C. Austin K. Rose G. M. Woodward D. J. (1999). Multiple single units and population responses during inhibitory gating of hippocampal auditory response in freely-moving rats. *Brain Res.* 825 75–85. 10.1016/S0006-8993(99)01187-7 10216175

[B42] Nagahara A. H. McGaugh J. L. (1992). Muscimol infused into the medial septal area impairs long-term memory but not short-term memory in inhibitory avoidance, water maze place learning and rewarded alternation tasks. *Brain Res.* 591 54–61. 10.1016/0006-8993(92)90977-H 1446233

[B43] O’Keefe J. Nadel L. (1978). *The Hippocampus as a Cognitive Map.* Oxford: Oxford University Press.

[B44] Pang R. Turndorf H. Quartermain D. (1996). Pavlovian fear conditioning in mice anesthetized with halothane. *Physiol. Behav.* 59 873–875. 10.1016/0031-9384(95)02137-X 8778880

[B45] Park S. Lee J. Park K. Kim J. Song B. Hong I. (2016). Sound tuning of amygdala plasticity in auditory fear conditioning. *Sci. Rep.* 6:31069. 10.1038/srep31069 27488731 PMC4973267

[B46] Phillips D. P. Irvine D. R. (1979). Acoustic input to single neurons in pulvinar-posterior complex of cat thalamus. *J. Neurophysiol.* 42 123–136. 10.1152/jn.1979.42.1.123 219155

[B47] Pinault D. (1996). A novel single-cell staining procedure performed in vivo under electrophysiological control: morpho-functional features of juxtacellularly labeled thalamic cells and other central neurons with biocytin or Neurobiotin. *J. Neurosci. Methods* 65 113–136. 10.1016/0165-0270(95)00144-1 8740589

[B48] Raybuck J. D. Lattal K. M. (2011). Double dissociation of amygdala and hippocampal contributions to trace and delay fear conditioning. *PLoS One* 6:e15982. 10.1371/journal.pone.0015982 21283812 PMC3023765

[B49] Robinson S. Bucci D. J. (2012). Fear conditioning is disrupted by damage to the postsubiculum. *Hippocampus* 22 1481–1491. 10.1002/hipo.20987 22076971 PMC3290706

[B50] Romanski L. M. LeDoux J. E. (1992a). Bilateral destruction of neocortical and perirhinal projection targets of the acoustic thalamus does not disrupt auditory fear conditioning. *Neurosci. Lett.* 142 228–232. 1454221 10.1016/0304-3940(92)90379-l

[B51] Romanski L. M. LeDoux J. E. (1992b). Equipotentiality of thalamo-amygdala and thalamo-cortico-amygdala circuits in auditory fear conditioning. *J. Neurosci.* 12 4501–4509. 1331362 10.1523/JNEUROSCI.12-11-04501.1992 PMC6575992

[B52] Ruusuvirta T. Korhonen T. Penttonen M. Arikoski J. (1995). Hippocampal evoked potentials to pitch deviances in an auditory oddball situation in the rabbit: no human mismatch-like dependence on standard stimuli. *Neurosci. Lett.* 185 123–126. 10.1016/0304-3940(94)11240-J 7746502

[B53] Sacchetti B. Lorenzini C. A. Baldi E. Tassoni G. Bucherelli C. (1999). Auditory thalamus, dorsal hippocampus, basolateral amygdala, and perirhinal cortex role in the consolidation of conditioned freezing to context and to acoustic conditioned stimulus in the rat. *J. Neurosci.* 19 9570–9578. 10531459 10.1523/JNEUROSCI.19-21-09570.1999 PMC6782906

[B54] Sacco T. Sacchetti B. (2010). Role of secondary sensory cortices in emotional memory storage and retrieval in rats. *Science* 329 649–656. 10.1126/science.1183165 20689011

[B55] Sakurai Y. (1990). Hippocampal cells have behavioral correlates during the performance of an auditory working memory task in the rat. *Behav. Neurosci.* 104 253–263. 10.1037/0735-7044.104.2.253 2346620

[B56] Sakurai Y. (2002). Coding of auditory temporal and pitch information by hippocampal individual cells and cell assemblies in the rat. *Neuroscience* 115 1153–1163. 10.1016/S0306-4522(02)00509-2 12453487

[B57] Steward O. (1976). Topographic organization of the projections from the entorhinal area to the hippocampal formation of the rat. *J. Comp. Neurol.* 167 285–314. 10.1002/cne.901670303 1270625

[B58] Syka J. Masterton R. B. (1988). *Auditory Pathway Structure and Function.* New York, NY: Springer 10.1007/978-1-4684-1300-7

[B59] Tehovnik E. J. Sommer M. A. (1997). Electrically evoked saccades from the dorsomedial frontal cortex and frontal eye fields: a parametric evaluation reveals differences between areas. *Exp. Brain Res.* 117 369–378. 10.1007/s002210050231 9438704

[B60] Tranel D. Brady D. R. Van Hoesen G. W. Damasio A. R. (1988). Parahippocampal projections to posterior auditory association cortex (area Tpt) in Old-World monkeys. *Exp. Brain Res.* 70 406–416. 10.1007/BF00248365 3384041

[B61] Turner J. G. Hughes L. F. Caspary D. M. (2005). Divergent response properties of layer-V neurons in rat primary auditory cortex. *Hear. Res.* 202 129–140. 10.1016/j.heares.2004.09.011 15811705

[B62] Vinnik E. Antopolskiy S. Itskov P. M. Diamond M. E. (2012). Auditory stimuli elicit hippocampal neuronal responses during sleep. *Front. Syst. Neurosci.* 6:49. 10.3389/fnsys.2012.00049 22754507 PMC3384222

[B63] Vinogradova O. S. (1975). “Functional organization of the limbic system in the process of registration of information: facts and hypotheses,” in *The Hippocampus* eds Isaacson R. L. Pribram K. H. (Boston, MA: Springer) 3–69.

[B64] Wang N. Gan X. Liu Y. Xiao Z. (2017). Balanced noise-evoked excitation and inhibition in awake mice CA3. *Front. Physiol.* 8:931. 10.3389/fphys.2017.00931 29209230 PMC5702325

[B65] Webster D. B. Popper A. N. Fay R. R. (1992). *The Mammalian Auditory Pathway: Neuroanatomy.* New York, NY: Springer 10.1007/978-1-4612-4416-5

[B66] Weinberger N. M. Gold P. E. Sternberg D. B. (1984). Epinephrine enables Pavlovian fear conditioning under anesthesia. *Science* 223 605–607. 10.1126/science.6695173 6695173

[B67] Winer J. A. Morest D. K. (1983). The medial division of the medial geniculate body of the cat: implications for thalamic organization. *J. Neurosci.* 3 2629–2651. 10.1523/JNEUROSCI.03-12-02629.1983 6655503 PMC6564649

[B68] Winer J. A. Schreiner C. E. (2011). *The Auditory Cortex.* New York, NY: Springer 10.1007/978-1-4419-0074-6

[B69] Wu G. K. Arbuckle R. Liu B. H. Tao H. W. Zhang L. I. (2008). Lateral sharpening of cortical frequency tuning by approximately balanced inhibition. *Neuron* 58 132–143. 10.1016/j.neuron.2008.01.035 18400169 PMC2447869

[B70] Xiong X. R. Liang F. Li H. Mesik L. Zhang K. K. Polley D. B. (2013). Interaural level difference-dependent gain control and synaptic scaling underlying binaural computation. *Neuron* 79 738–753. 10.1016/j.neuron.2013.06.012 23972599 PMC3755964

[B71] Zhang G. W. Sun W. J. Zingg B. Shen L. He J. Xiong Y. (2018). A non-canonical reticular-limbic central auditory pathway via medial septum contributes to fear conditioning. *Neuron* 97 406.e4–417.e4. 10.1016/j.neuron.2017.12.010 29290554 PMC5798467

[B72] Zhang H. Lin S. C. Nicolelis M. A. (2011). A distinctive subpopulation of medial septal slow-firing neurons promote hippocampal activation and theta oscillations. *J. Neurophysiol.* 106 2749–2763. 10.1152/jn.00267.2011 21865435 PMC3214118

