# Supplementary material for: Septal and Hippocampal Neurons Contribute to Auditory Relay and Fear Conditioning
Source: Front Cell Neurosci. 2018 Apr 16;12:102. doi: 10.3389/fncel.2018.00102 (PMC5911473; doi:10.3389/fncel.2018.00102)
Supplement: Supplementary file 1 [file Image_1.PDF]

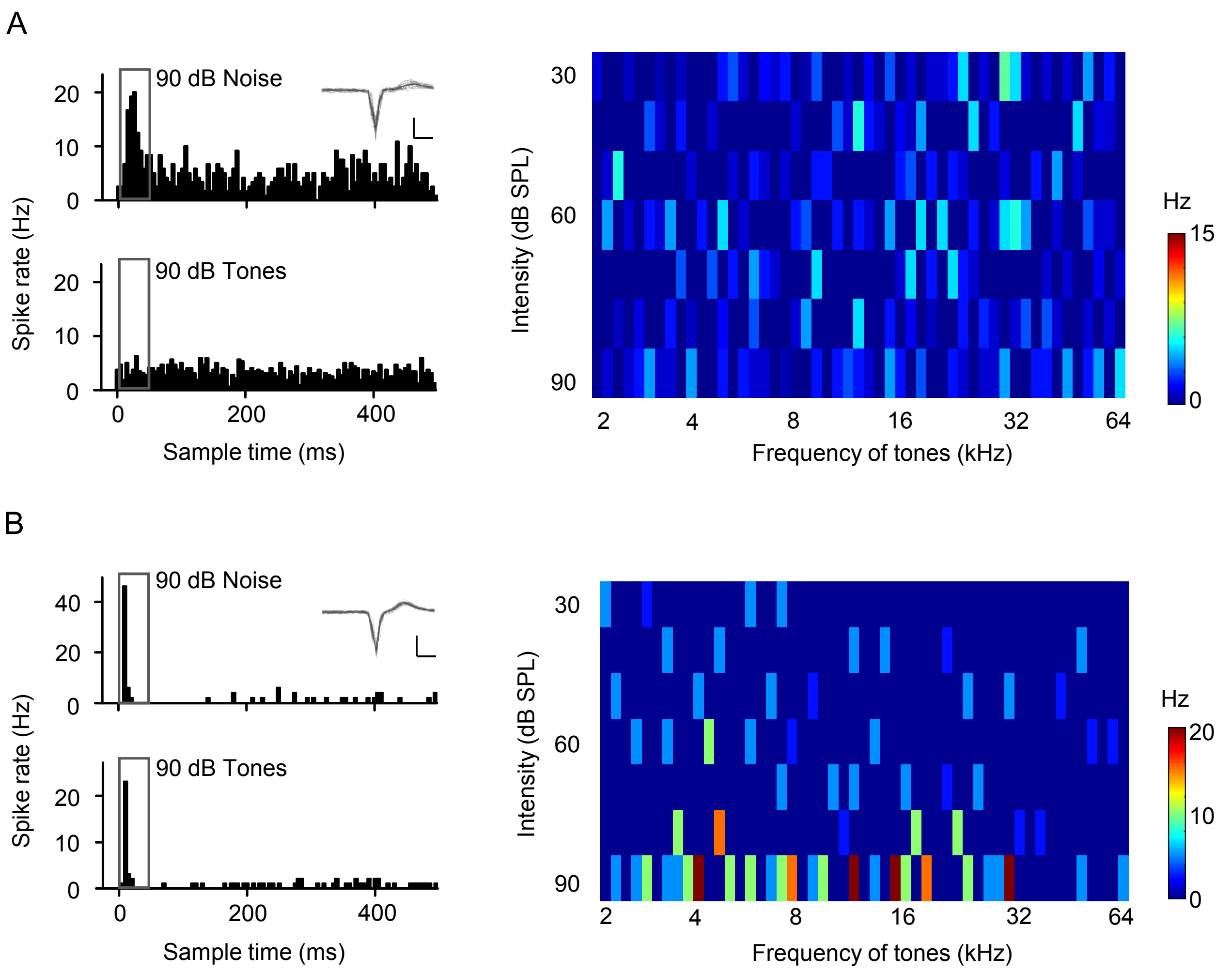

Supplemental Figure 1

Two examples of EC neurons respond to noise and tone stimuli.

(A) Left, spike responses PSTHs of an example EC neuron respond to white noise (the upper panel) and not to tone bursts (the lower panel). Right, Color map depicts spike rates in response to tones of different frequencies and intensities. Inset: superimposed 20 randomly selected spike waveforms. Scale: 40 pA, 0.5 ms. **(B)** Left, spike responses PSTHs of an example EC neuron respond to white noise (the upper panel) and tone bursts (the lower panel). . Inset: superimposed 20 randomly selected spike waveforms. Scale: 40 pA, 0.5 ms. Right, Color map depicts spike rates in response to tones of different frequencies and intensities, no clear TRFs were observed. Grey box indicates the duration of sound stimulation (50 ms).
