# Supplementary material for: Septal and Hippocampal Neurons Contribute to Auditory Relay and Fear Conditioning
Source: Front Cell Neurosci. 2018 Apr 16;12:102. doi: 10.3389/fncel.2018.00102 (PMC5911473; doi:10.3389/fncel.2018.00102)
Supplement: Supplementary file 3 [file Image_3.PDF]

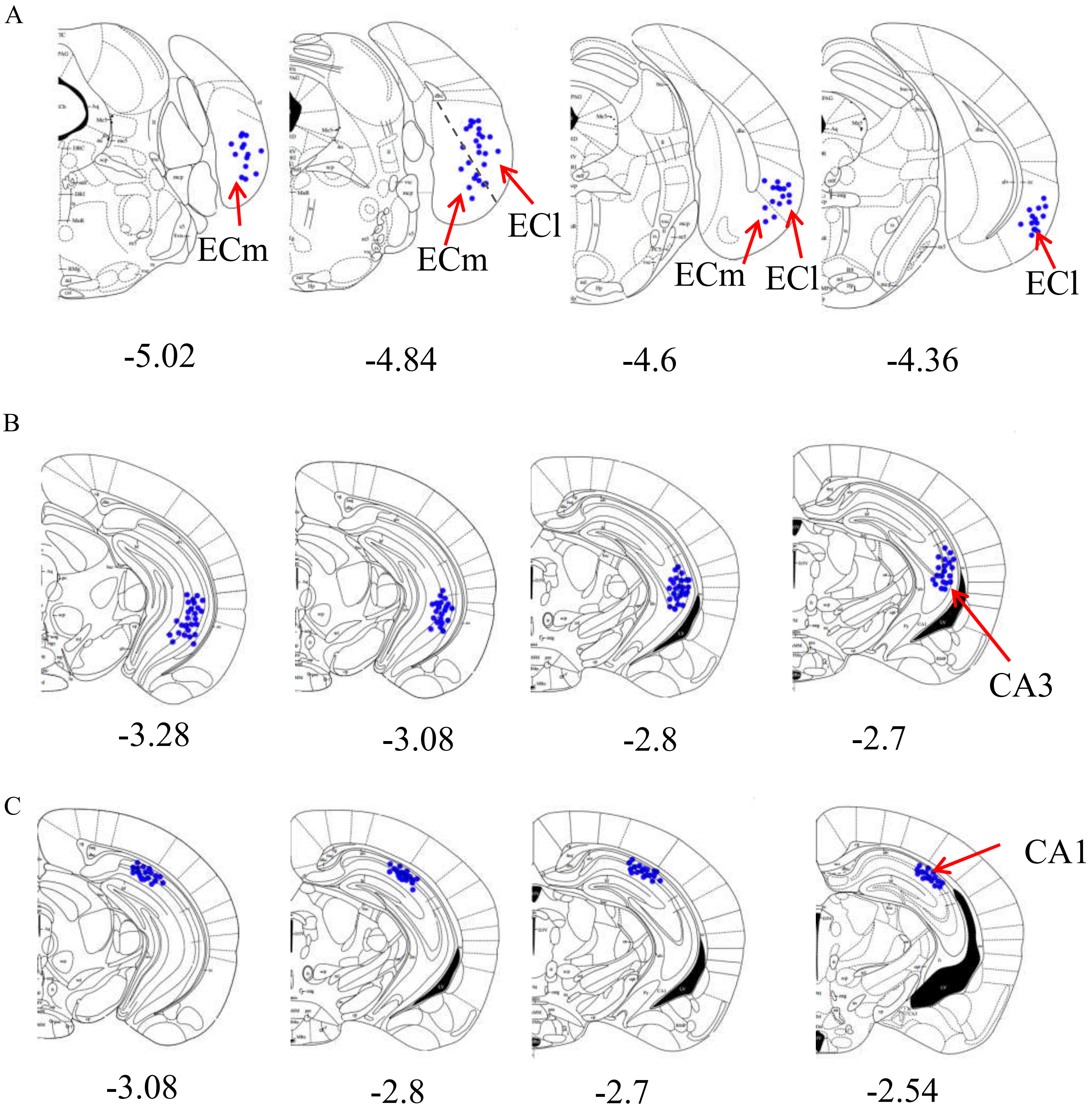

Supplemental Figure 3

(A-C) Schematic drawings show the reconstructed recording sites in EC (n= 65 cells), CA3 (n= 93 cells) and CA1 (n=69 cells). All of these neurons are auditory-responsive population. ECl, lateral entorhinal; ECm, medial entorhinal cortex.
