# Supplementary material for: Septal and Hippocampal Neurons Contribute to Auditory Relay and Fear Conditioning
Source: Front Cell Neurosci. 2018 Apr 16;12:102. doi: 10.3389/fncel.2018.00102 (PMC5911473; doi:10.3389/fncel.2018.00102)
Supplement: Supplementary file 4 [file Image_4.PDF]

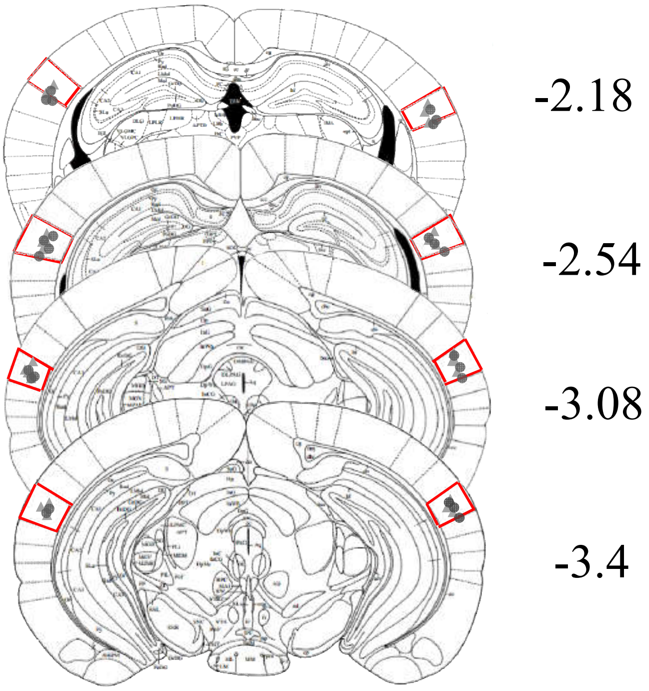

Supplemental Figure 4

Sites of saline (●) and muscimol (▲) cannula hits for auditory trace fear conditioning experiment. Red lines show the position of A1.
