# Supplementary material for: Septal and Hippocampal Neurons Contribute to Auditory Relay and Fear Conditioning
Source: Front Cell Neurosci. 2018 Apr 16;12:102. doi: 10.3389/fncel.2018.00102 (PMC5911473; doi:10.3389/fncel.2018.00102)
Supplement: Supplementary file 5 [file Image_5.PDF]

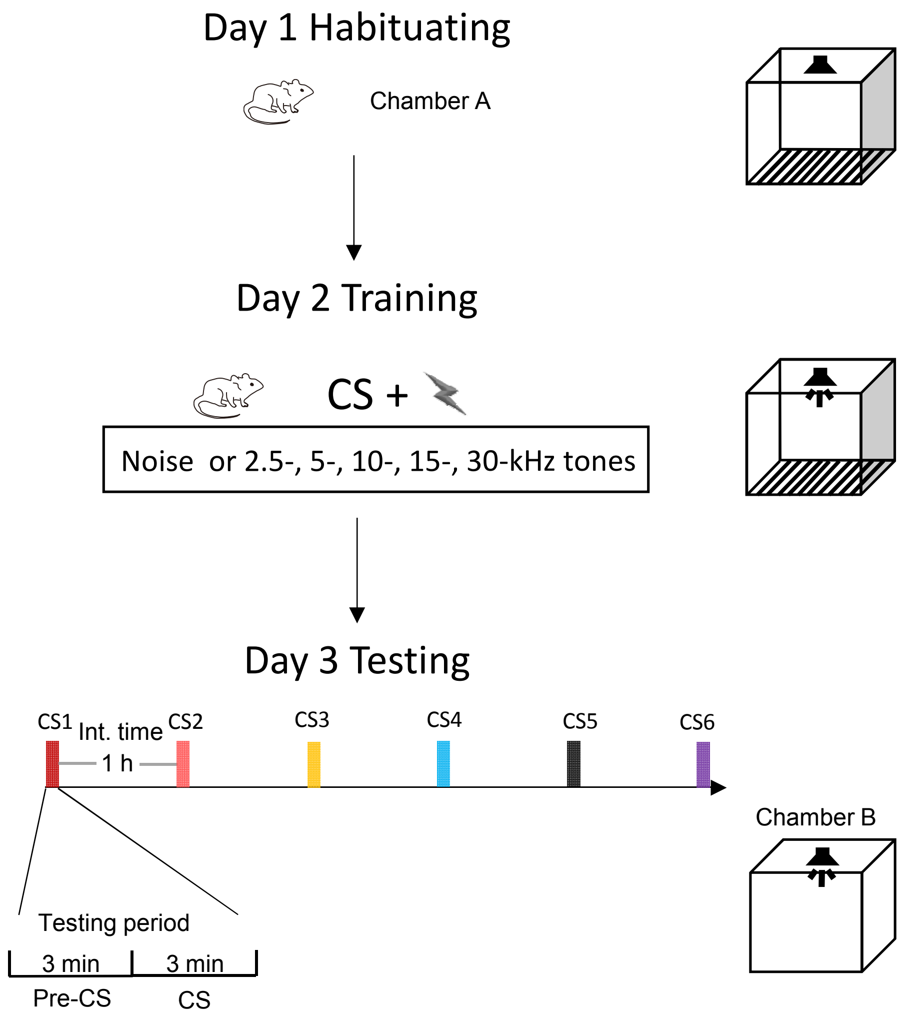

Supplemental Figure 5

Experimental design for sound characteristic conditioning. Auditory fear conditioning experiments included habituating, training and testing.

Each mouse was conditioned to one kind of auditory stimulus (no-sound, noise, 2.5-, 5-, 10-, 15-, or 30 -kHz tones) but tested with multiple sounds (noise, 2.5-, 5-, 10-, 15-, and 30- kHz tones) 24 h after the training. Each mouse underwent six different tests. The interval between each test was one hour.
